# Microfluidics for detection of exosomes and microRNAs in cancer: State of the art

**DOI:** 10.1016/j.omtn.2022.04.011

**Published:** 2022-04-27

**Authors:** Seyed Mojtaba Mousavi, Seyed Mohammad Amin Mahdian, Mohammad Saeid Ebrahimi, Mohammad Taghizadieh, Massoud Vosough, Javid Sadri Nahand, Saereh Hosseindoost, Nasim Vousooghi, Hamid Akbari Javar, Bagher Larijani, Mahmoud Reza Hadjighassem, Neda Rahimian, Michael R. Hamblin, Hamed Mirzaei

**Affiliations:** 1 Department of Neuroscience and Addiction Studies, School of Advanced Technologies in Medicine, Tehran University of Medical Sciences, Tehran, Iran; 2 Department of Pharmaceutical Nanotechnology, Faculty of Pharmacy, Tehran University of Medical Sciences, Tehran, Iran; 3 Endocrinology and Metabolism Research Center, Endocrinology and Metabolism Clinical Sciences Institute, Tehran University of Medical Sciences, Tehran, Iran; 4 School of Medicine, Kashan University of Medical Sciences, Kashan, Iran; 5 Student Research Committee, Kashan University of Medical Sciences, Kashan, Iran; 6 Department of Pathology, School of Medicine, Center for Women’s Health Research Zahra, Tabriz University of Medical Sciences, Tabriz, Iran; 7 Department of Regenerative Medicine, Cell Science Research Center, Royan Institute for Stem Cell Biology and Technology, ACECR, Tehran 1665659911, Iran; 8 Infectious and Tropical Diseases Research Center, Tabriz University of Medical Sciences, Tabriz, Iran; 9 Pain Research Center, Neuroscience Institute, Tehran University of Medical Science, Tehran, Iran; 10 Department of Applied Cell Sciences, School of Advanced Technologies in Medicine, Tehran University of Medical Sciences, Tehran, Iran; 11 Research Center for Cognitive and Behavioral Sciences, Tehran University of Medical Sciences, Tehran, Iran; 12 Iranian National Center for Addiction Studies (INCAS), Tehran University of Medical Sciences, Tehran, Iran; 13 Faculty of Pharmacy, Tehran University of Medical Sciences, Tehran, Iran; 14 Brain and Spinal Cord Research Center, Imam Khomeini Hospital, Tehran University of Medical Sciences, Tehran, Iran; 15 Endocrine Research Center, Institute of Endocrinology and Metabolism, Iran University of Medical Sciences (IUMS), Tehran, Iran; 16 Laser Research Centre, Faculty of Health Science, University of Johannesburg, Doornfontein 2028, South Africa; 17 Research Center for Biochemistry and Nutrition in Metabolic Diseases, Institute for Basic Sciences, Kashan University of Medical Sciences, Kashan, Iran

**Keywords:** microRNA, exosomes, microfluidics, biomarkers, cancer

## Abstract

Exosomes are small extracellular vesicles with sizes ranging from 30–150 nanometers that contain proteins, lipids, mRNAs, microRNAs, and double-stranded DNA derived from the cells of origin. Exosomes can be taken up by target cells, acting as a means of cell-to-cell communication. The discovery of these vesicles in body fluids and their participation in cell communication has led to major breakthroughs in diagnosis, prognosis, and treatment of several conditions (e.g., cancer). However, conventional isolation and evaluation of exosomes and their microRNA content suffers from high cost, lengthy processes, difficult standardization, low purity, and poor yield. The emergence of microfluidics devices with increased efficiency in sieving, trapping, and immunological separation of small volumes could provide improved detection and monitoring of exosomes involved in cancer. Microfluidics techniques hold promise for advances in development of diagnostic and prognostic devices. This review covers ongoing research on microfluidics devices for detection of microRNAs and exosomes as biomarkers and their translation to point-of-care and clinical applications.

## Graphical abstract

**Figure:**
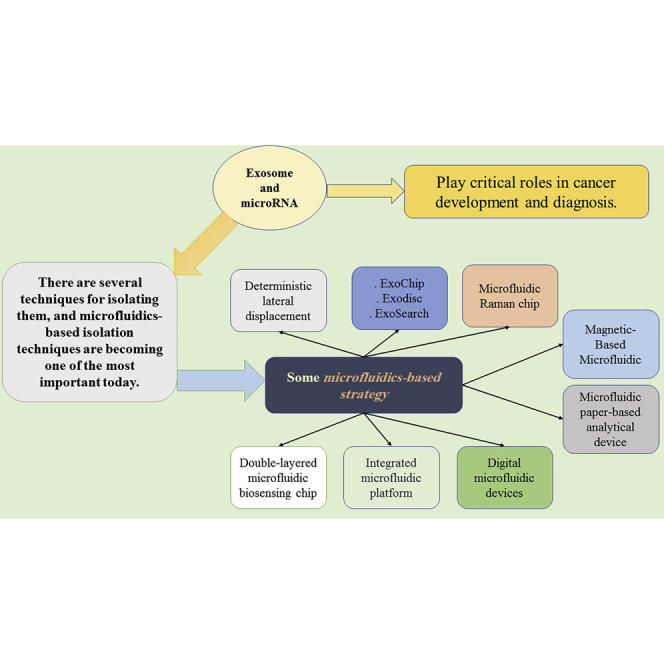

## Teaser

The emergence of microfluidics devices with increased efficiency in sieving, trapping, and immunological separation of small volumes could provide improved detection and monitoring of exosomes and micoRNAs involved in cancer.

## Introduction

Effective clinical diagnosis is necessary for early detection of cancer and monitoring tumor progression. Biomarkers and individual-specific molecular information have improved the process of clinical diagnosis and management of cancer in terms of identification, determination of disease burden and stage, and selection of the most effective treatment approach.^1^

Cancer contributes to a large proportion of global deaths each year. The International Agency for Research on Cancer reported an incidence rate of 18.1 million and a mortality rate of 9.6 million worldwide in 2017.^2^^,^^3^ Cancer is responsible for approximately one-sixth of all deaths worldwide, with an estimated 43.8 million people living more than 5 years after being diagnosed with cancer. Early, accurate, and economical diagnosis of cancer at molecular levels is critical to improve management, lower care costs, and boost therapeutic efficiency.^4^ Tissue biopsy, one of many forms of direct tumor biopsy, involves removing cells from the body with special needles or surgery and is a common step in standard clinical procedures for assessment of the tumor profile.^5^ The study of cancer cells and other cancer-related biomolecules, along with their microenvironment, using bodily fluids is an attractive approach to overcome the limitations of tissue biopsy to monitor disease progression. Over the last decade, several experimental studies have used analysis of body fluids, such as blood, serum, plasma, urine, saliva, cerebrospinal fluid, or pleural effusions, to investigate molecular cancer biomarkers.^6^^,^^7^ Also known as fluid biopsy, liquid biopsy is identification of tumor-derived components by sampling body fluids in a minimally invasive or non-invasive manner.^8^

Exosomes are small extracellular vesicles composed of a lipid bilayer membrane containing membrane-specific proteins that are 30–150 nm in size.^9^ It is difficult to determine whether extracellular vesicles (EVs) are exosomes based only on their size. Confusion about the origin and nomenclature of EVs has spread throughout the literature because vesicles with the same size as exosomes but that bud from the plasma membrane have also been called exosomes.^10^^,^^11^ The vesicle size distribution typically associated with exosomes is a 30- to 150-nm diameter, but their identity is based largely on their distinctive cup-shaped morphology seen in transmission electron microscopy images. However, much broader size distributions have been reported from some laboratories, and spherical vesicles with the morphological properties of exosomes and diameters up to ∼200 nm have been directly observed in cryogenic transmission electron microscopy (cryo-TEM) images.^11^, ^12^, ^13^

Various exosomal cargos (e.g., DNA, messenger RNAs [mRNAs], microRNAs [miRNAs], and proteins) are protected from degradation by the lipid bilayer membrane. Exosomes can be found circulating freely in some body fluids, which can be employed as clinical samples.^14^ Exosomes are therefore appealing candidates for designing new cancer detection methods.^15^^,^^16^ miRNAs, which are non-coding, single-stranded, endogenous small RNA molecules found in most eukaryotic organisms, can be also used to detect alterations in normal cell pathways.^17^ miRNAs are involved in post-transcriptional regulation of their target genes and have been shown to participate in regulation of more than 30% of the human genome and in almost all fundamental cell processes.^18^^,^^19^ miRNA expression patterns are often altered during normal developmental stages and in pathological conditions such as senescence, cardiovascular disease, and cancer.^20^^,^^21^ When miRNAs are encapsulated in EVs or attached to special lipid proteins, they are protected from RNase digestion and can be detected in plasma or serum in a remarkably stable manner.^22^^,^^23^ As a result, these small molecules are promising biomarkers used in fluid biopsies for cancer detection.^22^

First introduced as a biological tool in the early 1990s, microfluidics is now a field of study that manipulates microliter volumes in microchannels ranging in size only from 1–1,000 μm. Fluid flow is thus purely laminar, allowing precise monitoring of molecular concentrations.^24^^,^^25^ This integrative technique, which is now well recognized for controlling reagents in miniaturized devices, has progressed dramatically since then.^26^^,^^27^ Reduced sample size, less reagent consumption, rapid processing, increased sensitivity, automation, and real-time analysis are benefits of using microfluidics.^28^ One reason for using microfluidics methods in the life sciences is to adapt labor-intensive laboratory procedures in a manner similar to automated electronic circuits. The first microfluidics applications included electrophoresis on a chip, DNA microarrays, and polymerase chain reaction.^29^ After over a decade of progress in employing biosensors and single-cell assays, microfluidics-integrated devices have been expanded to include manipulation of RNA, proteins, and mammalian cells to improve diagnosis and prognosis. Biologic microfluidics devices can be employed in innovative formats to investigate more detailed cancer properties.

## Microfluidics and cancer

The Global Cancer Observatory (GLOBOCAN) 2018 reported about 18.1 million new cancer cases and 9.6 million cancer deaths in 2018 worldwide.^30^ Researchers have used many cancer screening, predictive determination, and tracking approaches to prevent and treat cancers. Few effective diagnostic techniques have been found so far that do not damage the affected individual to some extent during the diagnosis phase. Excessive ionizing radiation, for example in computed tomography (CT) scans, can pose possible health risks to the individual, especially at younger ages.^31^ Less intrusive methods, like ultrasound scans or magnetic resonance imaging (MRI), on the other hand, are considered insufficient for diagnosis of non-advanced or residual cancers.^32^^,^^33^ Tumor heterogeneity undermines the reliability of invasive “solid biopsy” procedures to monitor complex changes at the cellular level.^34^^,^^35^ Not only are these procedures invasive, but they can be inconvenient and expensive for the affected individual. In addition, people are more likely to be diagnosed at a later stage in the absence of regular screening. A late diagnosis can lead to worse treatment responses and lower 5-year survival rates.^36^ When it is necessary to continuously track the individual’s treatment response, improved cancer diagnosis methods should be less invasive and more affordable, allowing tumors to be detected and treated early. Exosomes and cellular RNAs have therefore received increasing attention in recent years for diagnosis of cancer and monitoring response to treatment.^37^

Conventional tests, such as polymerase chain reaction (PCR), enzyme-linked immunosorbent assay (ELISA), western blotting, immunofluorescence, flow cytometry, and immunodiffusion, have been used for biomarker identification in laboratory studies to diagnose infection, cancer, and other diseases.^38^^,^^39^ Most of these tests are complicated, lengthy, and sample consuming, depending on spacious laboratories and costly apparatus not available in deprived areas and developing countries. As a result, convenient, cheap, portable devices and methods are in high demand, especially point-of-care (POC) diagnostic devices that function well in resource-limited settings to diagnose and monitor pathological conditions. The spread of infectious diseases and the mortality of several cancers (e.g., oral, cervical, breast, and colorectal cancer) can be limited via POC devices providing rapid and early diagnosis. The acronym ASSURED (affordability, sensitivity, specificity, user-friendliness, rapid treatment, robust use, no special equipment, and delivery to patients) was introduced by the World Health Organization (WHO) as requirements for a diagnostic regimen in resource-limited populations.^40^

Microfluidics technology provides an expanding range of devices for management of small quantities of fluids that can be used for sensing and control of chemical, biological, and physical processes. Operational sensor construction can be integrated with the required electronic and optical elements using lithography-based manufacturing tools.^24^^,^^41^ The laminar flow characteristics observed in microfluidics flow contribute to maximal control over minimal volumes of sample fluid. Microfluidics platforms have many advantages, including enhanced reliability, sensitivity, accessibility, lower consumption of samples and reagents, reduced costs, quicker processing and response times, and the possibility of automated multiplexing.^42^, ^43^, ^44^ Despite these advantages, many challenges remain unsolved, and more advances will be needed toward employment and development of biomarker-based analytical devices. Considering the benefits of microfluidics, an increased interest has been shown in microfluidics use for biomarker discovery and sample evaluation to overcome the barriers and explore new possibilities.^45^

**Figure 1:**
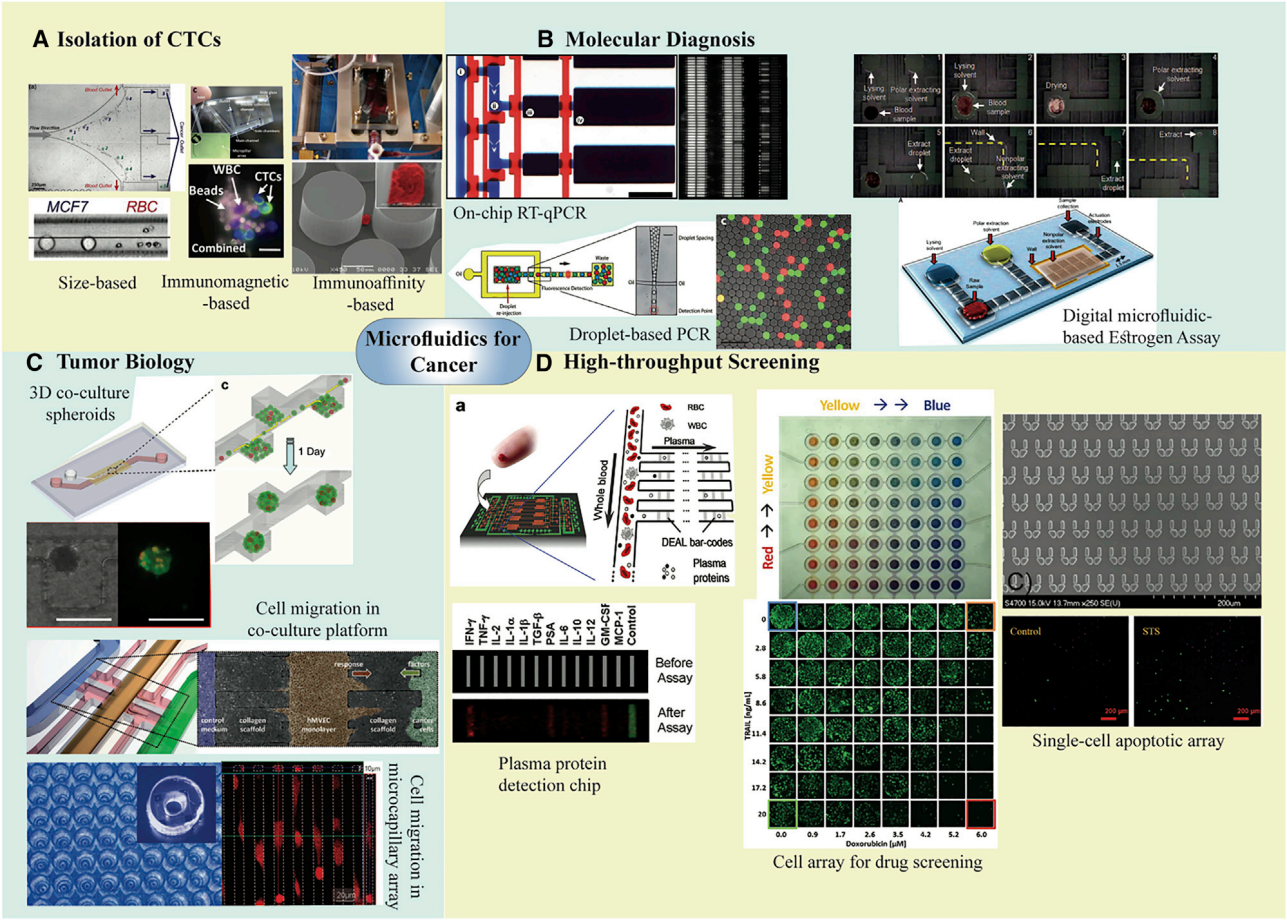
Microfluidics technology in cancer studies (A) Circular tumor cell (CTC) isolation by immunomagnetic-based, immunoaffinity-based, and size-based techniques. (B) Molecular diagnosis: droplet-based PCR for identifying rare mutations, on-chip single-cell qRT-PCR conducted in every reaction chamber, and droplet-scale estrogen assay for quantifying small amounts of tissues. (C) Tumor biology: migration of cancer cells in a micro-capillary array under mechanical confinement conditions, cell migration platform to explore the co-culture environmental effect, and generation of 3D co-culture spheroids for investigating the PCa metastatic microenvironment. (D) Programmable cell culture array for drug screening. High-throughput screening: an integrated blood barcode chip to identify plasma proteins and a single-cell array consisting of micromechanical traps for screening anti-cancer drugs that resulted in apoptosis. This figure was adapted from other studies.^46^, ^47^, ^48^, ^49^, ^50^, ^51^, ^52^, ^53^, ^54^, ^55^

A schematic overview of microfluidics technologies in cancer research is shown in Figure 1.

Biomedical researchers have produced multiple microfluidics devices that can analyze body fluids at micro scales for POC diagnosis and real-time evaluation.^56^ Global health is expected to be improved significantly by application of such devices, which are convenient for rural areas, developing nations, health care settings, and emergency applications. A plethora of diseases, including several cancer types (e.g., colorectal carcinoma, hepatocellular carcinoma, and ovarian and prostate cancer), various infections (e.g., food-borne disease, hepatitis B, meningitis, dengue virus, and coronavirus disease 2019 [COVID-19]), and conditions such as cardiovascular disease and Alzheimer’s disease can be detected by microfluidics devices at naturally occurring biomolecular concentrations.^56^, ^57^, ^58^, ^59^, ^60^, ^61^, ^62^, ^63^

## The biogenesis and function of exosomes

**Figure 2:**
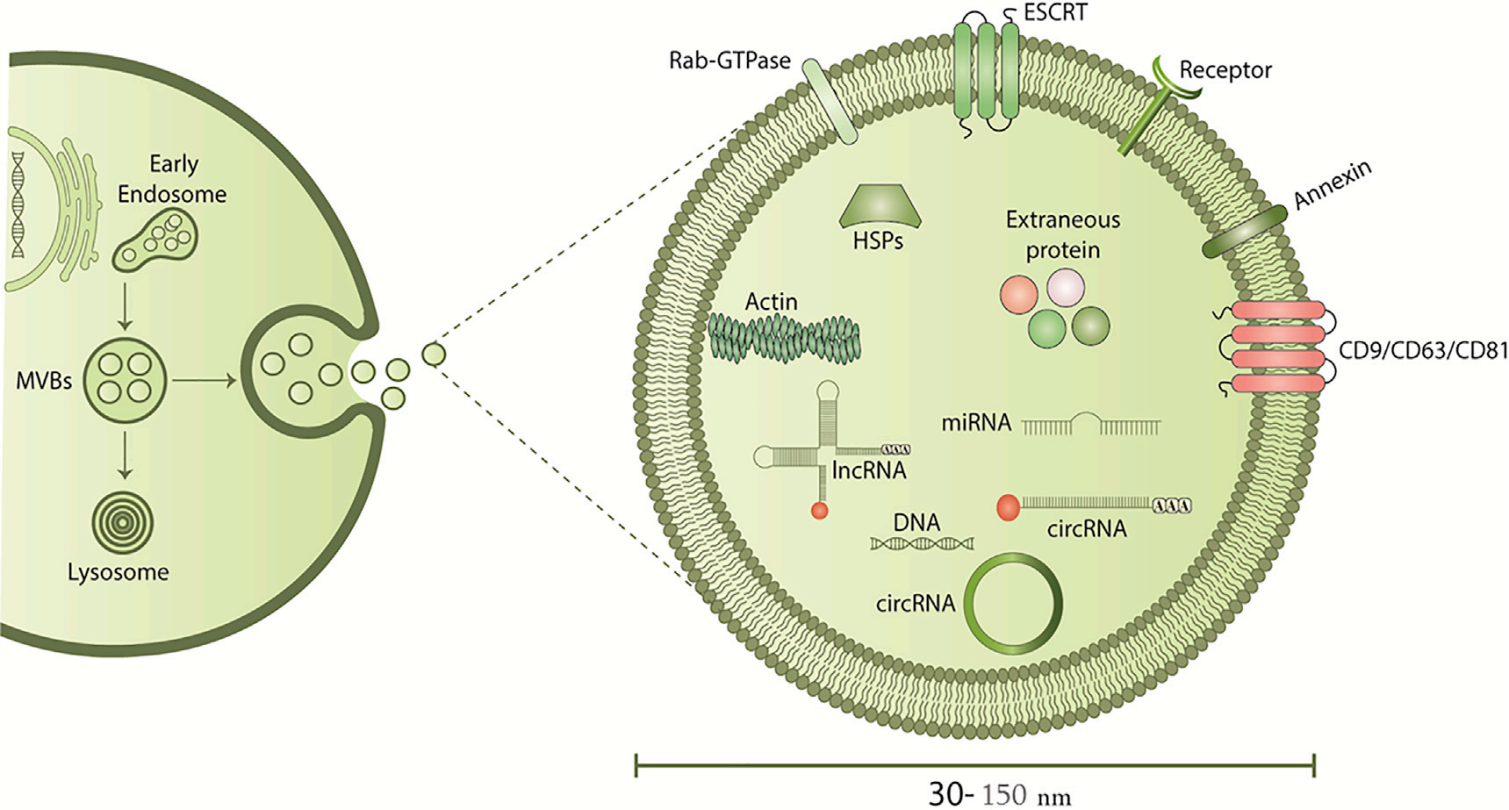
A schematic of biogenesis of exosomes and their cargos

Exosomes are small EVs with a lipid bilayer membrane containing membrane-specific proteins and are 30–150 nm in size (Figure 2).^9^^,^^64^ Exosomes can be distinguished from other EV types according to their biogenesis mechanisms. Exosomes are a subtype of EVs formed by an endosomal route. Exosomes are formed by intraluminal budding of multivesicular bodies (MVBs) leading to formation of intraluminal vesicles (ILVs), which are released into the extracellular medium when the MVBs and plasma membrane become fused. The main molecular pathway machinery for delivery and protein integration into exosomes membrane is the endosomal sorting complex required for transport (ESCRT), which is made up of four multi-protein complexes (ESCRT-0, -I, -II, and -III).^65^ ILV formation and exosome biogenesis are also modulated by ESCRT-independent processes. Lipids (ceramides), heat shock proteins, tetraspanins, and Rab proteins are involved in this pathway.^66^^,^^67^

Exosomes contain distinct proteins, lipids, and cellular payloads depending on their progenitor cell and biological origin. A common set of surface proteins, lipids, and nucleic acid payloads may, however, be seen in exosomes from diverse origins. The most common constituents of exosomes include tetraspanins (CD9, CD63, and CD81), lysosomal proteins (LAMP2B), heat shock proteins (HSC70), fusion proteins (CD9, GTPase, and Annexin), and MVB biogenesis-associated proteins (TSG101).^68^ Exosomes usually contain lipids derived from the parental cell plasma membrane and raft-associated lipids, such as cholesterol, phosphoglycerides, sphingolipids, and ceramides.^69^^,^^70^ An increased amount of phosphatidylserine is found in lipidic exosomes compared with their parental cells. The unique lipid and protein composition of exosomes provides their excellent physicochemical stability and allows direct fusion between the exosome and the recipient cell plasma membrane with no need for endosomal escape to deliver their cargo. The intracellular cargos comprise different kinds of biomolecules, including DNA and RNA molecules, which can deliver messages to recipient cells (Figure 2).^71^^,^^72^ Recent studies have demonstrated that exosomes can also transmit mitochondrial and chromosomal DNA between different cell types.^73^ Genetic materials, such as RNA and DNA molecules, can be transferred between neighboring or distant cells via exosomes.

**Figure 3:**
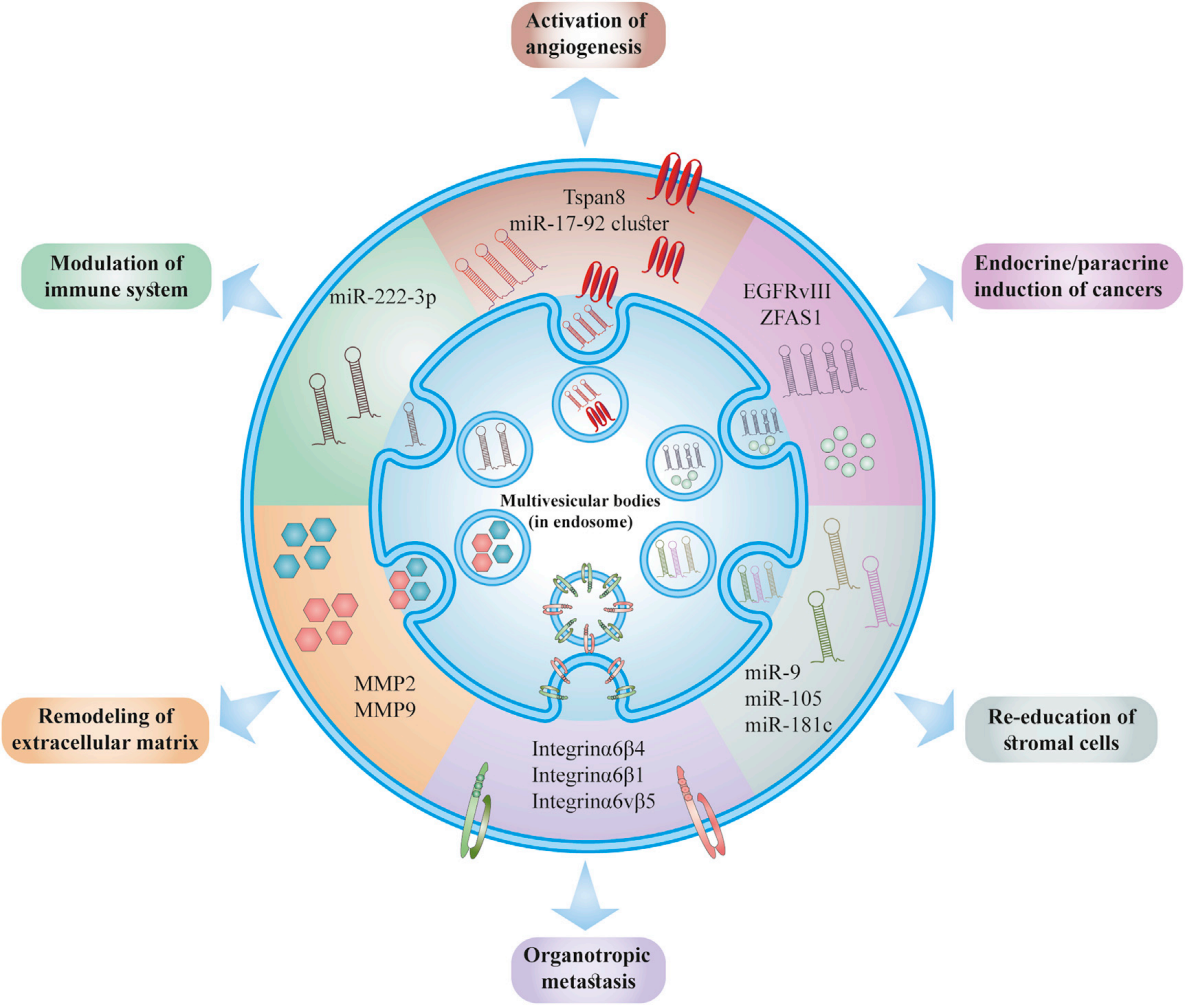
Summary of tumor-derived exosome-mediated functions Released exosomes from tumor cells modulate autocrine/paracrine induction of tumors and can induce angiogenesis, regulation of the immune system, re-education of stromal cells, organotropic metastasis, and remodeling the extracellular matrix. This figure was adapted from Tai et al.^79^

Exosomes play a critical role in regulation of a diverse range of physiological and pathological processes via horizontal delivery of biomolecules from progenitor to recipient cells. Exosomes carry out specific receptor-ligand interactions on the cellular membrane via functional proteins and lipids and deliver their bioactive contents into the recipient cells. Raposo et al.^74^ discovered that B cells release exosomes that are able to present antigens and induce effector T cell responses, which sparked further research into the physiological functions of exosomes. The immune surveillance function of exosomes has stimulated many researchers with interests in immunotherapy.^75^^,^^76^ Several other exosome-regulated biophysiological processes, such as cell proliferation, tissue regeneration, angiogenesis regulation, atherosclerotic plaque formation, coagulation cascades, and homeostasis maintenance, have become topics of interest.^77^^,^^78^ Exosomes serve a pleiotropic role in numerous disease mechanisms as important intercellular messengers *in vivo*. The modulatory effects of exosomes in tumor biology are the most often investigated pathogenic function of exosomes. Induction of tumor angiogenesis, tumor immunosuppression, and creation of pre-metastatic niches are among the mechanisms by which tumor-released exosomes can lead to the spread of malignancies (Figure 3).^80^ Exosomes can also play a role in delivery of neurodegeneration-related molecules and development of neurodegenerative disorders.^81^ For instance, β-amyloid peptides responsible for progression of Alzheimer’s disease are transported by exosomes and consequently deposited in particular brain regions.^82^ Exosomes are involved in release and transfer of toxic α-synuclein (αsyn), which forms aggregates that affect neuronal cells and cause brain pathology.^83^ Several other diseases, such as inflammatory conditions, cardiovascular diseases, virus infections, autoimmune diseases, diabetes mellitus, and, most importantly, cancers are also mediated by exosomes.^84^, ^85^, ^86^

## Exosomes and cancer: role in diagnosis and therapy

### Role of tumor-derived exosomes in cancer progression

Among the diverse functions of exosomes, activities associated with cancer pathology have received the most attention. Exosomes released by tumor cells attach to their target cells, promoting primary tumor formation, activating angiogenesis, stimulating stromal fibroblasts, promoting cancer cell adhesion to the extracellular matrix, forming a premetastatic niche, and suppressing the host immune response. Exosomes can also encourage their recipient tumor cells to avoid cell death and resist cytotoxic treatment through secretion of anti-apoptotic proteins.^87^^,^^88^ Peinado et al.^89^ revealed the potential of tumor-associated exosomes produced by mouse melanoma cells to promote melanoma formation in bone marrow cells. Disrupted formation and differentiation and abnormal function of hematopoietic cells are characteristic of bone marrow changes mediated by exosomes.^90^^,^^91^

Tumors are highly dependent on their blood supply, which provides cancer cells with abundant nutrients and oxygen to continuously grow in size.^92^^,^^93^ The new blood supply is provided by the balance between pro-angiogenic factors and anti-angiogenic factors becoming biased in favor of the former.^92^ In addition to many soluble factors, such as vascular endothelial growth factor (VEGF), exosomes have been found to be involved in determining the angiogenesis rate.^94^ When hypoxia is detected by tumor cells, they secrete their associated exosomes containing pro-angiogenic factors into the extracellular matrix to ensure an adequate supply of oxygen via the new vessels.^94^^,^^95^ Exosomes containing hypoxia-induced proteins are taken up by normal endothelial cells, which release their exosomal contents to stimulate formation of new tubules and, subsequently, a typical network of new blood vessels found in tumors.^96^ Several growth factors and cytokines are synthesized and secreted through the phosphatidylinositol 3-kinase (PI3K)/Protein kinase B (AKT) pathway when hypoxic tumor exosomes are taken up by host endothelial cells. Exosomes also damage tight junctions via delivery of miR-105 to endothelial cells, which then increases vascular permeability.^97^^,^^98^

The development and spread of tumors is highly dependent on exosomes. Exosomes may transfer growth-promoting genes and boost proliferation, invasion, and migration of metastatic cancer cells. For example, exosomes expressing the epidermal growth factor receptor (EGFR) can promote liver metastasis in an animal model.^99^ Exosomal miRNAs have also been discovered to be capable of down-regulating their downstream genes, which can drive cancer progression and spread to unaffected areas.^100^ Exosomes are also involved in cancer cell evasion from the immune response, leading to further proliferation or invasion. Exosomes can induce tumors to lower expression of phosphatase and tensin homolog deleted from chromosome 10 (PTEN), a tumor suppressor, especially in the brain and enhance brain metastasis.^101^^,^^102^ Another study reported that PTEN protein could be directly released from cells in exosomes and transferred to recipient cells, where it could restore its function.^103^ Exosomes have also been shown to restrict calcium absorption by non-tumor cells by releasing miRNA-122 to provide themselves with adequate glucose. Exosomes also have the ability to induce angiogenesis.^104^^,^^105^ In one study, tumor cells released exosomal tetraspanin (Tspan8), a key angiogenesis modulator, functioning via VEGF-independent pathways to stimulate endothelial cell proliferation and migration.^106^

Cancer cell exosomes can exert a bilateral effect on the immune system, stimulating immune cell activity in some cases and promoting tumor evasion from the immune system in other cases, such as nasopharyngeal cancer. This is possible because the exosomes facilitate CD4 T cell conversion and boost Treg recruitment to suppress anti-tumor immunity.^107^ Wen et al.^108^ used fluorescently tagged exosomes to show that exosomes can reduce immune activity by directly suppressing T cell proliferation and inhibiting natural killer (NK) cell cytotoxicity. Mizyazaki et al.^109^ showed that T cell-targeted exosomes can enhance immunological escape through induction of EBAG9 (estrogen receptor-binding fragment-associated antigen 9) expression. Antitumor immunity has been shown to be greatly boosted when tumor cells are exposed to heat stress. A potential anticancer vaccine could be designed based on the heat-mediated increase of immunogenicity of carcinoembryonic antigen (CEA)+ cancer cells.^102^

Treatment of several cancers, especially breast cancer, is often disrupted by frequent development of drug resistance in cancer cells during therapy. Drug resistance has been partly linked to the activity of exosomes and EVs.^110^^,^^111^ Exosomes have been shown to increase the interactions between stromal cells and breast cancer cells (MDA-MB-231). Exosomes released from drug-resistant tumor cells have been found to affect antiviral RIG-I (retinoic acid-inducible gene 1 enzyme) signaling and can be transferred to recipient tumor cells to increase drug resistance by affecting the NOTCH3 pathway.^112^^,^^113^ The process of exosomal transfer has been proposed to be mediated by an increase in stromal cell-induced RAB27B and exosomal 5′tripohosphate RNA-mediated activation of RIG-I signaling. Non-transformed MRC5 human diploid fibroblasts (stromal cells) were injected into MDA-MB-231 xenograft female nude mice to confirm the study results. STAT1 (signal transducer and activator of transcription 1) expression was consequently upregulated, resulting in less cell death and faster disease progression *in vivo*.^114^

### Tumoral exosomes as cancer biomarkers

Body fluids like blood from individuals with cancer are rich in tumor-derived exosomes, which partly reproduce the molecular and genetic composition of the tumor cells.^115^^,^^116^. Much attention has been directed toward possible application of such exosomes as a “liquid biopsy” for non-invasive detection of cancer.^117^^,^^118^ Exosomes have shown good potential as cancer biomarkers in personalized medicine because of their easy accessibility and ability to recapitulate the properties of their parental cells.^119^ Differences in contents and membrane composition have been observed in exosomes released from cells under various conditions (Table 1).^148^, ^149^, ^150^ The exosomal contents, derived from tumor cells, include a variety of nucleic acids, lipids, proteins, and biomarkers that may be a valuable source of diagnostic, prognostic, and therapeutic information.^151^

**Table 1:** Exosomal molecular markers in various cancers

Cancer type	Exosomal molecular marker	Reference
Colorectal	CPNE3	Sun et al.^120^
Colorectal	miR-1246, miR-23a, miR-21, miR-150, let-7a, miR-223, miR-1224-5p, miR-1229	Ogata-Kawata et al.^121^
Colorectal	CRNDE-h	Liu et al.^122^
Colorectal	miR-21	Uratani et al.^123^
Gastric	lncUEGC1, lncUEGC2	Lin et al.^124^
Gastric	HOTTIP	Zhao et al.^125^
Gastric	ZFAS1	Pan et al.^126^
Gastric	miR-423-5p	Ouyang et al.^127^
Pancreatic	miR-191, miR-451a, miR-21	Goto et al.^128^
Pancreatic	GPC1	Melo et al.^129^
Pancreatic	miR-17-5p	Que et al.^130^
Pancreatobiliary tract	miR-1246, miR-4644	Machida et al.^131^
Liver	hnRNPH1	Xu et al.^132^
Liver	LINC00161	Sun et al.^133^
Liver	ENSG00000258332.1, LINC00635	Xu et al.^134^
Pancreatic	hTERT	Goldvaser et al.^135^
Lung	MALAT-1	Zhang et al.^136^
Lung	14-3-3ζ	Sun et al.^137^
Ovarian	ephrinA2	Li et al.^138^
Ovarian	miR-200a, miR-200b, miR-200c	Meng et al.^139^
Ovarian	miR-21, miR-100, miR-320	Pan et al.^140^
Prostate	miR-125, miR-19	Bryzgunova et al.^141^
Prostate	SAP30L-AS1	Wang et al.^142^
Prostate	ADIRF	Øverbye et al.^143^
Prostate	LincRNA-p21	Işın et al.^144^
Melanoma	exo-MIA, exo-S100B	Alegre et al.^145^
GBM (glioblastoma)	RNU6	Manterola et al.^146^
Bladder	TACSTD2	Chen et al.^147^

Each cancer type displays different exosomal compositions. For example, 100% of exosomes from individuals with pancreatic cancer show glypican-1 (GPC1+) expression compared with only 2.3% in healthy individuals. The specificity and sensitivity of this biomarker for pancreatic cancer have been found to be relatively high, even at early stages.^129^ Another exosomal marker, macrophage migration inhibitory factor (MIF), has been shown to be related to the chance of liver metastasis in individuals with stage I pancreatic cancer.^80^ Estimation of exosomal vimentin levels after gemcitabine treatment provides prognostic information for individuals with pancreatic cancer.^152^ For example, exosomal miR-103 has been shown to be upregulated in hepatocellular carcinoma (HCC) tumors and suggested to be able to affect normal endothelial cells to encourage angiogenesis; it could therefore be a possible future HCC biomarker.^153^ lnc-sox2ot, Long non-coding RNAs (lncRNA)-activated in renal cell carcinoma with sunitinib resistance (ARSR), lnc-h19, and some other lncRNAs have been detected in exosomes in the circulation and have been successfully correlated with tumor stage and survival rate.^125^^,^^154^^,^^155^ Hence, exosomes could be highly sensitive and specific biomarkers for cancer detection and prediction of metastasis in the future, and exosomal miRNAs play a critical role in cancer metastasis and may be an effective therapeutic target for cancer therapy.^156^^,^^157^

Exosomal analysis still has some challenges, the most important of which is the low levels of exosomes in bodily fluids. To obtain a sufficient concentration of exosomes, a relatively large amount of body fluid has to be extracted. These drawbacks mean that full-scale investigations of exosomal biomarkers are challenging, leaving much of the potential unexplored.

## Exosome isolation methods

Exosomes must be accurately separated from a broad mixture of cells, proteins, and unwanted components to permit characterization and analysis of these unique EVs. Exosome isolation methods should be highly efficient and be able to isolate exosomes from a variety of different sample compositions. Multiple optical and non-optical approaches have been designed to assess the quality of isolated exosomes, including measuring their size, size distribution, biochemical composition, shape, and abundance.^158^ To isolate exosomes at sufficient numbers and concentrations, many different approaches have been tested as a result of rapid technological breakthroughs occurring in the laboratory. To enhance exosome isolation, each methodology takes advantage of a unique property of exosomes, such as density, surface proteins, size, and shape. Each exosome isolation method has its own set of benefits and drawbacks. Ultracentrifugation-based isolation techniques, immuno-isolation techniques employing magnetic microbeads, and extraction kits have been employed. Microfluidics-based techniques for purification and analysis of exosomes can include biosensing and basic proteomics in a single system.^159^

Although some reviews have previously covered similar topics,^160^, ^161^, ^162^, ^163^ in the present study we discuss microfluidics-based techniques not only for exosome analysis but also for detection of miRNAs, which has not been accomplished previously.

**Figure 4:**
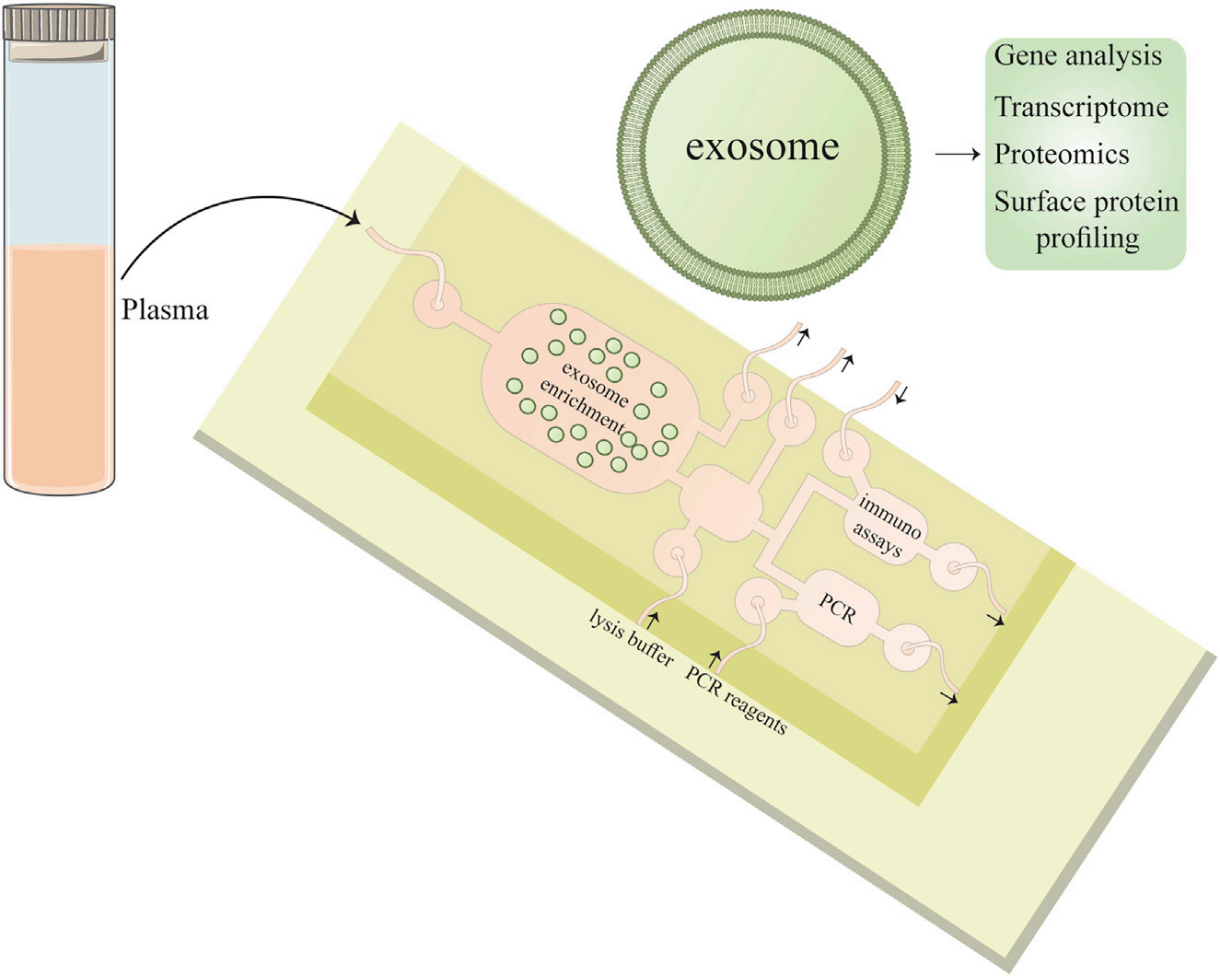
Novel and conventional techniques for exosome isolation Conventional techniques of EV isolation are as follows: differential ultracentrifugation (dUC) and size-exclusion chromatography (SEC). SEC uses a porous stationary phase against biofluids as a mobile phase to elute the molecules differentially with an opposite speed relation to their size. That is, at first, larger particles will elute, continued by smaller vesicles. Because smaller vesicles will pass into and flow via the pores, it results in a longer route and time of elution. dUC is based on EV subpopulation separation using slowly increasing acceleration rates. Novel exosomal methods are as follows. Polyethylene glycol (PEG)-based precipitation applies a solution to promote polymer-entrapped vesicle aggregation in a more significant number. The immunoaffinity (IA) capture method involves antibodies targeted for exosomal surface proteins to isolate specific vesicle populations. Chips with specific antibody-mediated binding are applied by microfluidics (MF) technology to efficiently capture the exosomes. Ultrafiltration (UF) relies on a filter with a particular pore size that specifically produces a vesicle-rich filtrate to the desired size. This figure was adapted from Sidhom et al.^164^

Some conventional as well as novel methods of exosome isolation are shown in Figure 4.

### Ultracentrifugation

Ultracentrifugation is the most popular method for isolating exosomes; it involves multiple centrifugation stages: 800 × *g* for 10 min, 2, 000 × *g* for 10 min, and finally more than 100,000 × *g*.^100^^,^^165^ Complete vesicle precipitation highly depends on attaining a sufficiently high speed and a long duration (more than 16 h) of centrifugation. Exosomes are usually separated from biofluids usually using higher-speed centrifuges; i.e., 10,000–20,000 ×*g*. Jeppesen et al.^166^ used a centrifuge with ultrahigh speed at 100,000–200,000 ×*g* to separate exosomes from cell culture in the presence of 20% fetal bovine serum (FBS) after 16 h. However, differential centrifugation leads to sediments containing exosomes as well as other EVs and contaminating protein debris, which are responsible for unsatisfactory results and relatively low yields.^166^^,^^167^

Classic ultracentrifugation can be complemented with a sucrose gradient centrifugation step to improve the purification process.^165^ Although exosomes move upward in a sucrose gradient, proteins form aggregates and sediments in this approach. Melo et al.^129^ utilized the sucrose gradient ultracentrifugation method to separate exosomes expressing tumor cell surface proteoglycan glypican-1 (GPC-1). Individuals with pancreatic or breast cancer have been shown to have GPC-1-bearing exosomes. GPC-1 exosomes can effectively allow differentiation of individuals with pancreatic ductal adenocarcinoma (PDAC) from healthy individuals even at early stages.^129^

The miRNA contents of exosomes, which are similar to their cells of origin, have prompted researchers to investigate exosomal miRNA expression levels as biomarkers, using ultracentrifugation for exosome isolation.^168^, ^169^, ^170^, ^171^, ^172^ Lee et al.^173^ recently employed the ultracentrifugation technique for quantitative detection of miR-21 in exosomes from breast cancer cells using a molecular beacon fluorescent oligonucleotide probe. Although ultracentrifugation proved more effective in allowing miR-21 hybridization, other commercial exosome isolation methods (e.g., Total Exosome Isolation and ExoQuick-TC) used in this study also led to accurate detection results.^37^

### Magnetic microbeads

Exosome separation from cell culture medium or from bodily fluids using antibody-coated magnetic beads is a simple, quick, and promising technique that has been claimed to show better recovery and higher purity than ultracentrifugation.^174^ Use of bead-exosome complexes has the benefit of allowing flow cytometry to quickly characterize the exosome surface phenotype.^165^ Only partial recovery of total exosomes is possible with this procedure because only some particular types of exosomal membrane surface proteins direct bind to monoclonal antibodies; therefore, the beads are attached to only a limited number of exosomes. Several cancer types, such as ovarian, colon, and lung cancer, have been studied with immuno-isolation of exosomes via magnetic microbeads.^175^^,^^176^ The adhesion molecule epithelial cellular adhesion molecule (EpCAM), which is overexpressed on epithelial progenitor cells, carcinoma cells, and cancer-initiating cells and can also be present in exosomes, has been targeted with this method.^176^ Tauro et al.^174^ used a colorectal cancer cell line, LIM1863, to analyze affinity capture mechanisms for exosomes compared with differential centrifugation and density gradient centrifugation. The affinity capture method managed to enrich exosomal markers and exosome related proteins at least two times better than the other two exosome isolation techniques. Affinity-based approaches, according to Klein-Scory et al.,^177^ are more successful to detect and isolate cancer-specific biomarkers along with other proteins expressed in exosomes. They found that EpCAM-based affinity exosome purification differed in efficiency depending on the heterogeneity of the pancreatic cancer cells (Paca44 and Panc1) and the surface proteins expressed on exosomes.

### Extraction kits

The ExoSpin Exosome Purification Kit (Cell Guidance Systems, USA), Invitrogen Total Exosome Isolation Kit (Life Technologies, USA), ExoEasy Maxi Kit (QIAGEN, Germany), and ExoQuick exosome precipitation solution (BioCat, Germany) are among the commercial kits used frequently by research groups to perform exosome isolation. These kits typically require an overnight incubation period at 4° C in polyethylene glycol or other polymers and lead to exosome sedimentation using low-speed centrifugation (10,000–20,000 × *g*).^178^ These techniques are attractive for exosome isolation because they are reasonably rapid and do not demand a time-consuming ultracentrifugation step. Some challenges remain when using such kits; for instance, they may sediment non-vesicular particles in the same way as ultracentrifugation, and their mode of action has yet to be completely established. High cost is another drawback of these kits, especially for high-throughput processing of large numbers of samples.^179^

Several studies have attempted to detect cancer biomarkers in exosomes using commercial isolation kits. For instance, Bryant et al.^180^ utilized the ExoMiR extraction kit (Bio Scientific, Austin, TX, USA) to comprehensively analyze the miRNA profile in exosomes. miR-141 miR-375 levels were found to be correlated with metastatic prostate cancer (PCa), and miR-107 and miR-574-3p concentrations were found to be higher in exosomes from individuals with PCa compared with healthy individuals. More extensive cohort studies are required to verify whether the miRNAs identified by this study can be used as biomarkers for early PCa diagnosis. In another study, serum exosome miR-21, precipitated by the ExoQuick kit, was found to be higher in individuals with esophageal squamous cell carcinoma (ESCC), suggesting its possible potential for detecting ESCC at primary stages.^181^ The ExoQuick kit was also used in another study in which exosomal miR-373 was found to be elevated in individuals with aggressive breast cancer.^182^ In this study, miR-373 serum levels were found to be higher in estrogen receptor (ER)-negative and progesterone receptor (PR)-negative cancer cell subtypes compared with receptor-positive ones, each subtype presenting a different prognosis. However, the clinical benefit of miR-373 is questionable because different levels of miR-373 were detected in ER/PR-negative and receptor-positive cases. ExoQuick kits more commonly allow precipitation of exosomes and early identification of cancer-related biomarkers compared with analyzing free miRNAs. Melanoma inhibitory activity (MIA), S100B, and tyrosinase-related protein 2 (TYRP2) have been identified in serum as exosomal diagnostic and prognostic biomarkers for melanoma. However, application of TYRP2 for early diagnosis of melanoma has not been firmly established.^145^

## Microfluidics-based isolation techniques

**Figure 5:**
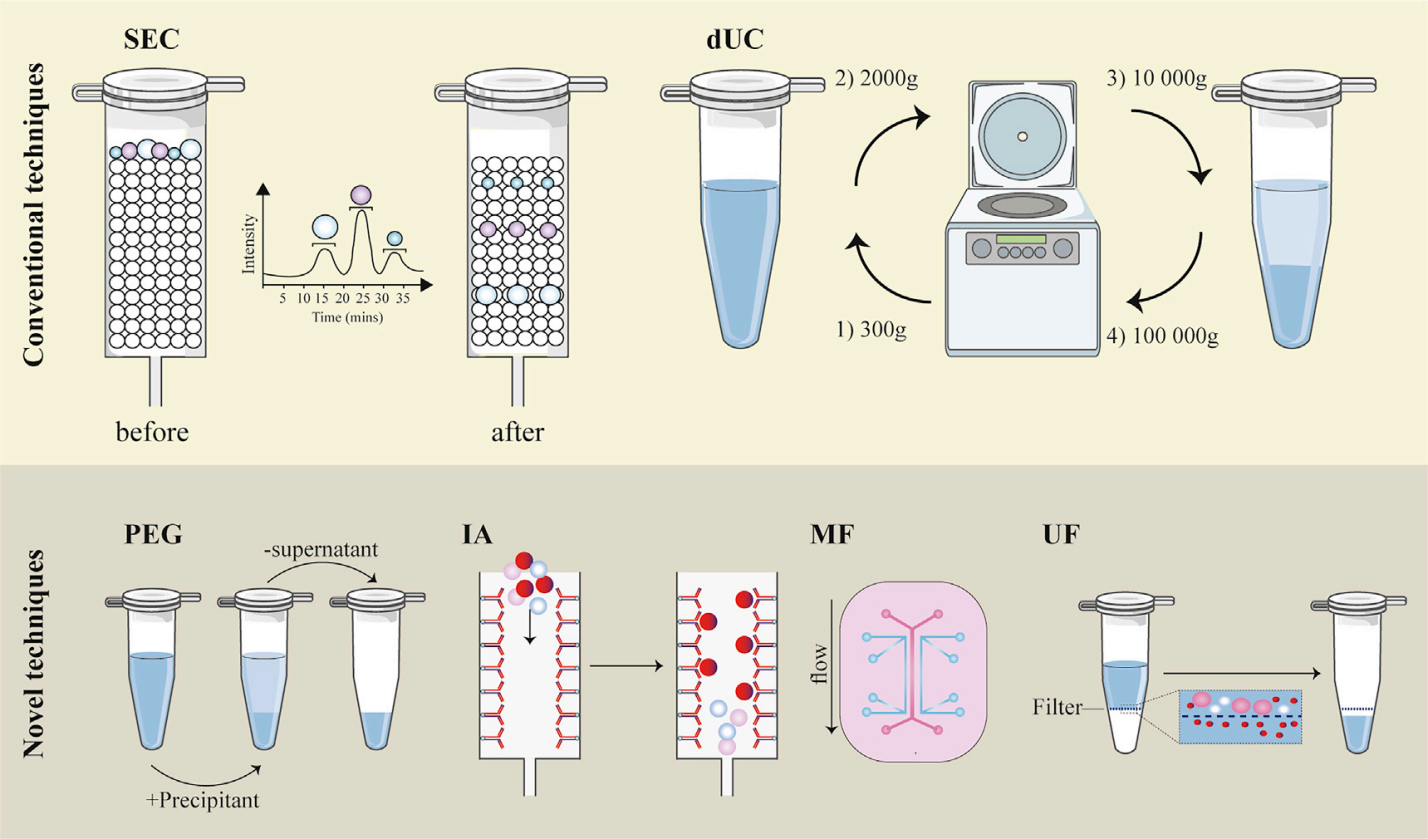
Illustration of an MF device for exosome analysis Plasma or serum has flows through an antibodiy-containing chamber that detects exosome surface proteins. Exosomes are captured in this chamber, and waste is piled up in an outlet. Retained exosomes are stained with various antibodies for profiling of surface protein. The exosomes can then be transferred to another chamber for lysis and deliver their cargos into various chambers. Proteins can be recognized by sandwich immunoassays, whereas RNA and DNA can be examined by DNA microarrays or PCR. Exosome cargo can be study off-chip for more molecular profiling. This figure was adapted from Garcia-Cordero et al.^183^

The design and preparation of microfluidics-based devices have immensely benefitted from remarkable breakthroughs in microfabrication technology, Microfluidics can now utilize the physical and biological features of exosomes at the microscale. Novel filtering processes, such as acoustic, electrophoretic, or electromagnetic manipulation, can be used in addition to conventional techniques based on size, density, or immunoaffinity.^159^ Required sample volume, reagent consumption, and isolation time can be noticeably decreased with adoption of such technologies. A schematic depiction of a microfluidics device for exosome analysis is shown in Figure 5.

### Exosome isolation by microfluidics

Common approaches to exosome isolation using microfluidics-based methods include size-based, immuno-affinity-based, and dynamic microfluidics manipulation. Nano-filters, nano-porous membranes, and nanoarray devices are types of separation systems that depend on the size of the exosomes. The main parts of a sandwich-like microfluidics device designed for size-based enrichment of exosomes in low sample volumes are a detachable membrane filter (with 0.1-μm pores), a microfluidics circuit located beneath the membrane, and two permanent ring magnets that work together to drive the filtered exosomes into the collection channel.^184^ The droplet size can be controlled in the range of 20–400 μm by varying the water and oil flow rates. The sandwich-like microfluidics device has the advantages of a short processing cycle, low cost, and low flow resistance.^185^ This type of device allows easy replacement of filter units during the process. Soft lithography is used to create the microfluidic device, which comprises three polydimethylsiloxane (PDMS) layers attached to a glass slide. Negative pressure is used to guide the fluid in the microfluidics channels, with mechanically activated valves placed in the PDMS layers. Non-cured PDMS is employed to adhere the PDMS layers together, and traditional O_2_ plasma activation is utilized to form an irreversible bond between the PDMS structure and the glass slidxe. Although, in most cases, blood samples are analyzed, other biofluids, such as serum or urine, can also be utilized by these microfluidics devices.

Exosomes have been isolated from small-volume samples by an acoustic nanofilter device using a contact-free continuous flow method. Surface acoustic waves (SAWs) are used to sort the EVs into different sizes and concentrations. A continuous flow setup with a low risk of coagulation is provided by the SAW methodology. The SAW generates a drag force dependent on the volume of the particle. Acoustic power and fluid speed are two additional parameters that can be adjusted to affect particle velocity. The method has a high yield and good separation resolution. The findings also showed that SAW can be a potentially rapid and successful method for isolating exosomes.^186^ The advantages of SAW microfluidics are simple fabrication, high biocompatibility, rapid fluid actuation, versatility, compact and inexpensive devices and accessories, contact-free particle manipulation, and compatibility with other microfluidics components.^187^ This technique has mostly been used for blood sample analysis with up to 50-μL sample volume. Fluid pumping in such a closed-loop chamber can be used to mimic the action of small blood vessels, making it useful for clinical applications and laboratory studies.^187^

Deterministic lateral displacement (DLD) is a continuous-flow microfluidics particle separation method discovered in 2004 that has been successfully applied for separation of blood cells, yeast, spores, bacteria, viruses, DNA, droplets, and more.^188^ DLD arrays are used to separate particles or cells with sizes ranging from millimeters to sub-micrometers.^189^ Researchers have reported high-resolution isolation of exosomes with sizes between 20 and 110 nm, using DLD arrays created in a microchannel.^190^ Huang et al.^191^ first introduced the nano-DLD technique to regulate sample flow through nanopillar arrays in rows with a pre-determined gap, pitch, and diameter. To extend the range of potential applications, the specific arrangement of geometric features in DLD has also been adapted and/or coupled with external forces (e.g., acoustic, electric, and gravitational) to separate particles on the basis of other properties, such as shape, deformability, or the dielectric properties of particles.^192^ Another study showed that a nano-pillar array with a spacing of 25 nm can be generated. A SiO_2_ mask was used to shape the nano-pillars with an aspect ratio (depth/gap) of 10:1 in silicon using an optimized deep reactive ion etching (RIE) method3,3' -dioctadecyloxacarbocyanine, perchlorate (DiO). Scanning electron microscopy (SEM) has been used to assess the extent to which samples have a uniform size.^193^

**Figure 6:**
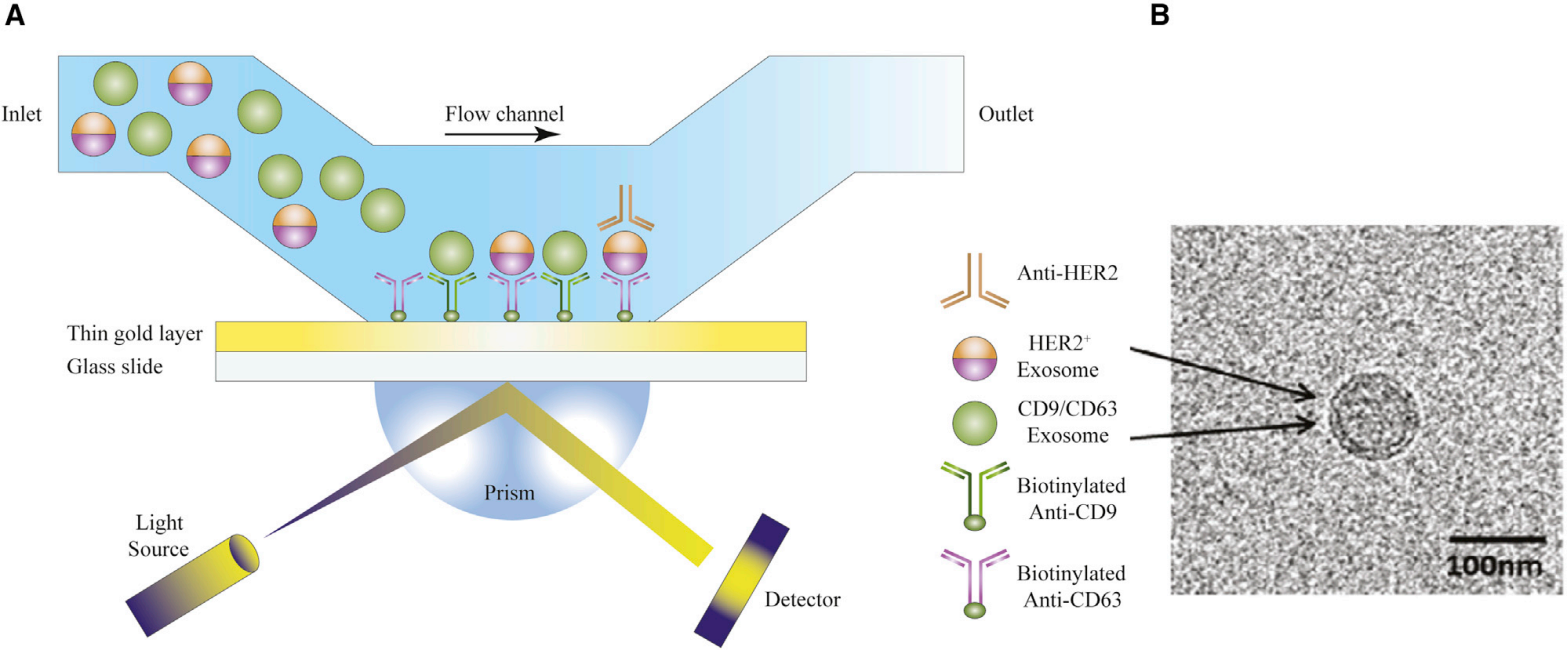
Experimental strategy for exosome immobilization and characterization using ExoChip (A) Schematic of the exosome capture and analysis procedure using ExoChip. The blood is collected for serum extraction from healthy or diseased individuals, and then exosomes are captured by flowing serum through a CD63 antibody-coated ExoChip. To visualize the captured exosomes, the ExoChip is processed for membrane-specific dye (DiO) staining. (B) The ExoChip is designed to measure the levels of fluorescently stained exosomes through fluorescence intensity measurements using microplate readers and allows molecular characterization of exosome contents through a variety of standard assays, including protein analysis (western blot) and mRNA/miRNA analysis (RT-PCR/miRNA open array). This figure was adapted from other studies.^194^^,^^195^

In another study, an immunoaffinity-based microfluidics system was used to selectively separate particles depending on unique biomarkers expressed on the exosome surface. Magnetic beads with appropriate antibodies to bind the exosomes can be manipulated in microchannels in these devices. The ExoChip is one example shown in Figure 6A.^194^ The ExoChip is a simple, low-cost microfluidics-based platform to isolate circulating EVs enriched in exosomes directly from blood or serum, allowing simultaneous capture and quantification of exosomes in a single device. The ExoChip is fabricated from PDMS and functionalized with antibodies against CD63, an antigen commonly overexpressed in exosomes. Subsequent staining with a fluorescent carbocyanine dye (DiO) that specifically labels the exosomes can quantify exosomes using a standard plate reader.^194^ This microfluidics system comprises one or more channels, each of which leads to numerous wells connected by a thin channel. Increased numbers and decreased sizes of the chambers are used to lower the fluid speed in the wells, facilitating exosome interaction with anti-CD63 antibodies (an overexpressed exosomal antigen) on the beads. The velocity gradient permits the sample to be mixed between the connecting channels. Soft lithography produces the PDMS structure, which is attached to glass via plasma treatment to create the microfluidics device.

Microfluidics systems can handle small liquid volumes because of their miniaturized channels and can conduct several procedures in parallel. When multifunctional nanostructures, such as nanopillars, nanowires,^196^ nanoparticles,^197^^,^^198^ graphene-based materials, or nanoporous layers^199^^,^^200^ are coupled with microfluidics channels, the separation in microfluidic channels is enhanced, holding promise for future design of nanostructure-coupled microfluidics systems.^186^^,^^194^^,^^198^^,^^201^^,^^202^

A polyethylene glycol (PEG)-lipid-modified surface was utilized initially to examine exosome isolation and microfluidics immobilization with no need for antibodies as additional approaches.^163^ A microfluidics system was introduced by Wang et al.^203^ for multi-scale filtration based on a ciliated nanowire-on-micropillar platform with the ability to trap exosome-like lipid vesicles. Routine microfabrication approaches are applied to achieve the array of micropillars, and subsequently the side walls of micropillars are etched via porous silicon nanowires. Next, liposomes are trapped selectively by the nanowire forest, and proteins and beads larger than 500 nm are filtered. Then the nanowires are dissolved in PBS for 24 h to recover the trapped liposomes. Concurrently, the exosomes are isolated by a three-dimensional nanowire network specific to DNA extraction with higher efficiency than ultracentrifugation.^204^ In a similar way, microfluidics equipment can be utilized to trap EVs exploiting PDMS-anchored ZnO nanowires. One of the most promising methods appears to be physical entrapment of exosomes by applying nanowires because of their great capture efficiency and fairly high throughput.^163^ Trapping based on pillars involves one or more arrays of closely spaced structures capable of ejecting larger molecules between structures. Such filters can reverse the flow for recovering exosomes. Multiscale biofluid filtration and exosome isolation have been carried out by an array of ciliated nanowire micropillars located in the microfluidics system.^203^ A range of 30–200 nm could be considered to adjust the distance between the nanowires of a nanowire array. Hence, a tool with suitable interstitial locations could trap exosomes without considering tiny molecules and larger components. The trapping procedure is fairly rapid (about 10 min) with admirable recovery efficiency (about 60%). High-purity trapped exosomes are likely to be released through porous nanowire dissolution in PBS. The effects of saturation could potentially reduce the recovery efficiency of the tool with a greater sample volume. Exosomes can be trapped on the microfluidics framework using a three-dimensional PDMS-anchored ZnO nanowire network.^205^ One of the most promising methods appears to be physical entrapment of exosomes by applying nanowires because of their great capture efficiency and high throughput compared with ultracentrifugation. Exosomes can possibly be extracted in combination with other microvesicles with similar surface properties, so there is a need for off-chip downstream analysis to detect specific exosomal proteins.^206^

Introduction of magnetic microbeads containing affinity ligands allows the exosomes to be isolated from serum (anti-CD63, anti-EGFR). After lysing the exosomes, the RNA is transferred to glass beads for further processing. A surface-enhanced Raman scattering (SERS)-based isolation approach has been reported for tumor-based exosome detection, either qualitatively or quantitatively. SERS signals can be detected in the presence of target exosomes and SERS nanoprobes. The exosomes are trapped by a sandwich-type immunoassay and magnetic-dependent precipitation of the nanobeads.^207^

Unfortunately, only exosomes rich in specific surface antigens can be separated using immunoaffinity-based separation microfluidics devices. Faster and easier exosome separation systems have been proposed by combining novel microfluidics techniques with dynamic approaches based on external forces, such as flow field-flow fractionation (FIFFF) or ultrasound. Davies et al.^208^ devised a microfluidics filtration technique to separate exosomes and extract mRNA from whole-blood samples instead of using antibody binding. Their technology eliminates the need for time-consuming centrifugation and affinity purification using antibodies. Vesicles may be differentiated from cells or detritus based on their size through use of a porous polymer monolithic membrane (PPM) attached to a poly(methyl methacrylate) (PMMA) chip. The exosomes can be selectively extracted and biological impurities rejected by changing the size of the membrane pores. An acoustic nanofilter device can distinguish EVs based on density and size with an exosome recovery rate of 80%.^186^ Despite the good purification and recovery rate of the separated subcellular elements in the samples, the FIFFF device is still considered a complicated nanostructure.^209^

### Exosome characterization by microfluidics

Biophysical, molecular, and microfluidics approaches are among the techniques used to characterize exosomes. Exosomal size range can be defined using biophysical approaches. Novel label-free biophysical approaches are rapidly emerging in exosome research, with the potential to revolutionize exosome (EXO) diagnostics. These methods include use of nanodevices for EXO purification, small-angle scattering (SAS) and diffraction, vibrational spectroscopy, scattering, and nanoindentation for characterization.^162^

Optical particle tracking is a biophysical approach to evaluate the size distribution of exosomes between 10 nm and 2 μm as well as their concentration. The velocity of the particles is assessed by monitoring the exosomal migration trajectories.^210^ Photon correlation spectroscopy, resistive pulse sensing, atomic force microscopy, FIFFF, cryoelectron microscopy, and TEM are other biophysical characterization techniques.^211^, ^212^, ^213^, ^214^, ^215^, ^216^, ^217^ Different versions of the Malvern Panalytical NanoSight instrument are routinely utilized.^215^^,^^218^^,^^219^ The size of blood exosomes has been quantified and analyzed through nanoparticle tracking analysis using a Malvern Instruments Nanosight NS500 device plus TEM with a Zeiss Libra 120 in a study conducted by Kapogiannis et al.^218^ In another study, Flaherty et al.^219^ employed a NanoSight NS300 (Malvern Instruments) and a ViewSizer 3000 (MANTA Instruments) to investigate the particle concentration and size of adipocyte-derived exosomes. Capello et al.^220^ used a Particle Metrix ZetaView Nanoparticle-tracking analytical instrument to evaluate exosome isolation.

Exosomal surface proteins can be categorized using molecular methods. Exosome size and shape in particular can be determined using flow cytometry.^221^ Flow cytometry and Raman spectroscopy (which uses laser light to produce a scattered infrared signal) are molecular approaches for exosome characterization. A variety of molecules, such as peptides and nucleic acids, can be detected by this method, which, in turn, characterizes the exosome chemical composition.^222^

Exosomal RNA contents have been studied using a variety of approaches, including microarray analysis, digital droplet PCR (ddPCR), and next-generation sequencing (NGS).^223^ miRNAs in exosomes derived from leukemic cells exposed to toluene have been quantified by microarray analysis to investigate changes in gene expression.^224^ NGS has also been used to assess the expression of exosomal miRNAs from human stem cells. ddPCR has been used to perform absolute quantification of miRNAs.^225^^,^^226^ Western blotting, proteomics technologies, and fluorescence-based cell sorting have also been used to examine the protein composition of exosomes.^184^^,^^227^^,^^228^

## Microfluidics and exosomes in cancer

Use of circulating exosomes to identify cancer and track response to therapy using continuous release of tumor-derived exosomes containing specific biomolecules is becoming more common.^11^^,^^64^^,^^115^^,^^229^ The technological challenges involved in isolating and analyzing these miniaturized vesicles containing different markers have severely limited their wider application.^11^

Kanwar et al.^194^ created a simple, affordable, microfluidics-based approach that could separate exosome-enriched circulating EVs (cirEVs) from healthy and pancreatic cancer blood samples with the aim to collect and quantify exosomes in a single device. They used ExoChip, an anti-CD63 antibody-dependent microfluidics device constructed from PDMS, to collect specific exosomes. Exosomes were quantified via a conventional plate reader after being labeled with a fluorescent carbocyanine dye (DiO). Exosomes were significantly higher from individuals with cancer (2.3-fold, p < 0.001) than those collected from healthy persons, according to 10 repeated ExoChip tests utilizing serum from five individuals with pancreatic cancer and five healthy persons. The ExoChip is claimed to be an appropriate platform for exosome-based diagnosis and research on human cancer molecular monitoring.^194^

Non-small cell lung cancer (NSCLC), accounting for over 85% of lung cancers, is a main global cause of cancer-related mortality, with a very low 5-year survival rate of only 15% (stage IIIA).^230^, ^231^, ^232^ Acquiring enough biopsy tissue for diagnosis is problematic, leading to the majority of individuals only being diagnosed at advanced non-resectable stages. Obtaining tissue samples before therapy is exceedingly challenging, which severely restricts the availability of histologic and molecular findings for personalized treatment.^233^ Therefore, researchers have attempted to detect serum biomarkers in serum from individuals with NSCLC. This involved selective isolation of exosomes followed by quantitative analysis of IGF-1R (type 1 insulin growth factor receptor) phosphorylation levels and IGF-1R total expression levels.^234^ However, clinical assessment of IGF-1R requires a highly invasive immunohistochemistry (IHC) test to be performed on the tumor biopsy tissue.^235^ He et al.^236^ described, for the first time, a combined microfluidics technique that allows direct on-chip immuno-isolation and *in situ* protein analysis of exosomes in plasma obtained from individuals with NSCLC. Exosome separation and enrichment, online chemical lysis, sandwich immunoassay with fluorescence detection, and protein immunoprecipitation are used in this approach. In addition, a cascade microfluidic circuit is constructed to optimize and accelerate the workflow for circulating exosome proteomic analysis. This method allows direct and highly sensitive isolation of specific exosome subpopulations from plasma samples and quantitative detection of surface and intravesicular markers in less than 100 min. This technique is used to conduct exosome subpopulation phenotyping with a collection of common exosomal and tumor-specific markers as well as multiparameter assessment of intravesicular biomarkers in the targeted subpopulation. Exosomes are used instead of invasive traditional tissue biopsies to evaluate IGF-1R total expression and phosphorylation levels in individuals with NSCLC. He et al.^236^ suggest that their microfluidics exosome analysis platform can serve as part of the essential infrastructure to advance exosome biology and clinical applications.

In women in the United States, ovarian cancer is the fifth most common cause of cancer-related death. It is most often detected at clinical stages III/IV, causing death in 80% of affected women within 5 years.^237^ There is currently no approved method for identifying CA125, the most often employed ovarian tumor biomarker, with acceptable sensitivity and specificity.^238^ Tumor exosomes found in body fluids like ascites and blood might be used as non-invasive biomarkers for monitoring and early detection.^239^^,^^240^. Zhang et al.^241^ studied changes in CD24, EpCAM, and FRα protein expression in ovarian cancer-derived exosomes to test whether a 3D-nanopatterned microfluidics device could perform non-invasive screening of cancer biomarkers. Circulating exosomes were found to contain detectable FRα concentrations in early-stage ovarian cancer serum samples using this approach. Exosomal FRα levels were considerably higher compared with normal samples, suggesting that this approach may be used to detect early-stage malignancies. These findings encourage future research into the clinical potential of exosomal FRα as a blood biomarker for sensitive and specific detection of ovarian cancer. Novel biosensing technologies can provide clinical diagnosis using this approach.^241^ Zhao et al.^240^ designed ExoSearch, a simple microfluidics platform that efficiently detects blood plasma exosomes for *in situ* immunomagnetic bead detection in a multifunctional manner. The consistent flow in the ExoSearch chip allows quantitative extraction and release of blood plasma exosomes in a broad range of volumes from 10 mL to 10 L. Using a training set of ovarian cancer plasma samples, they used the ExoSearch chip to perform simultaneous measurements of CA-125, EpCAM, and CD24 (three different exosomal tumor biomarkers) for blood-based ovarian cancer detection. The diagnostic power was estimated to be as high as that of a conventional test (a.u.c. = 1.0, p = 0.001). This finding is likely to open up new horizons for application of microfluidics for clinical cancer diagnosis and fundamental exosome studies.^240^

**Figure 7:**
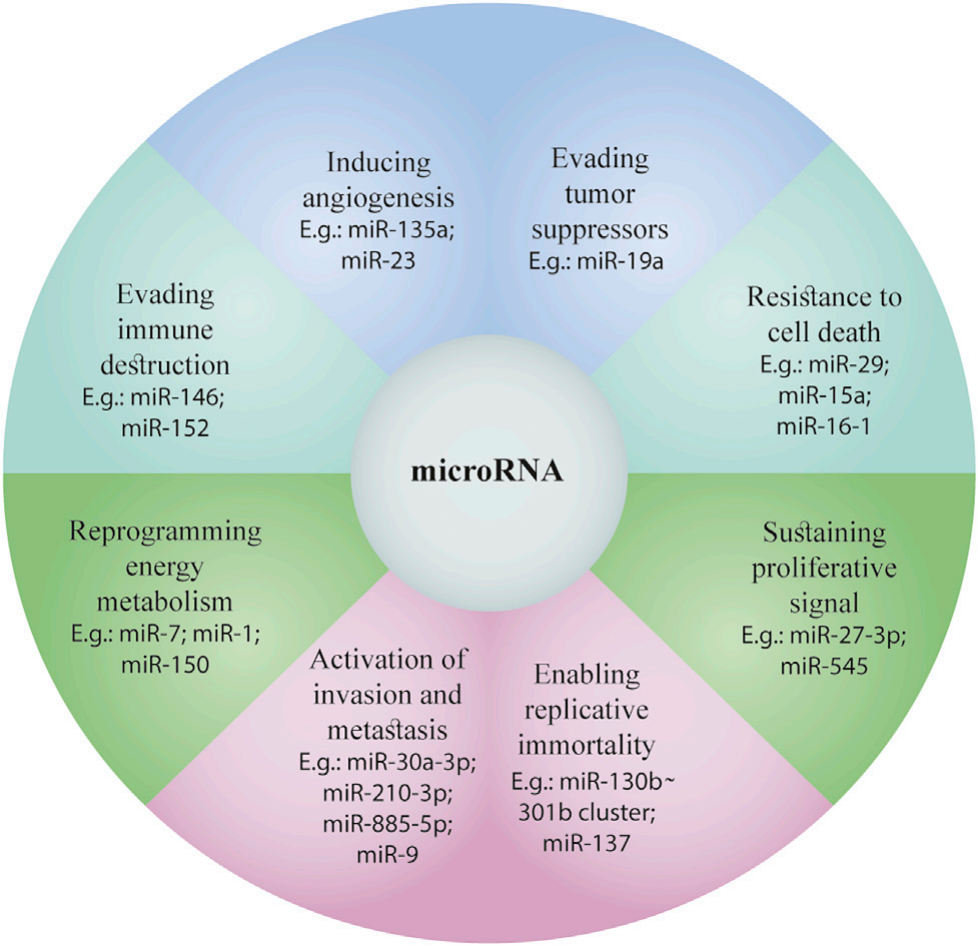
Down- or up-regulation of miRNAs contributes to the cancer-driving steps Often one miRNA affects more than one hallmark with one prevailing tissue-dependent mechanism. This figure was adapted from Detassis et al.^249^

The most common malignancy among women is breast cancer.^242^ Exosome levels in breast cancer serum have been reported to be considerably higher than in normal donor serum.^243^^,^^244^ Each stage and variant of breast cancer displays different combinations of EV proteins, which can be used as diagnostic markers of tumor progression or as general cancer diagnostic markers, akin to those used in fluid biopsy. As a result, HER2 molecular classification is critical when choosing the right approach for treatment of each individual with breast cancer. EpCAM expression has also been shown to be upregulated in a variety of carcinomas, such as breast cancer.^245^ Fang et al.^246^ designed a clinical microfluidics chip for immunocapture and evaluation of circulating exosomes in small sample volumes. Six individuals with breast cancer and three healthy control individuals were recruited for this study, which evaluated EpCAM-positive exosomes in the plasma samples. Compared with healthy controls, the affected individuals showed a substantial rise in EpCAM-positive exosome levels. Circulating HER2-positive exosomes were measured in 19 individuals with breast cancer for molecular categorization. The expression level of HER2, a breast cancer marker, is frequently measured in clinical trials using immunohistochemical labeling of tumor biopsy samples.^247^^,^^248^ The findings showed that expression levels of exosomal HER2 were almost identical to those measured by immunohistochemistry in tumor tissue. They suggested that microfluidics chips showed potential for diagnosis and molecular categorization of breast cancer.^246^ Sina et al.^195^ described a straightforward method for determining the fraction of clinically relevant exosomes (CREs) in the bulk exosome population extracted from breast cancer blood samples. The percentage of CREs can provide information about the stage of the cancer and allow non-invasive screening of expression levels of breast cancer receptors, which vary depending on the individual (Figure 7). The CRE proportion is determined using a surface plasmon resonance (SPR) platform in a two-step procedure. The first step is initial separation of the bulk exosome population using CD9 and CD63 (two tetraspanin biomarkers), and the second step is detection of CREs in the collected bulk exosomes using HER2 tumor-specific antibodies. Concentrations around 2,070 exosomes/μL can be measured in breast cancer cell cultures by this relatively sensitive approach, with 14%–35% of tumor-specific exosomes present in breast cancer serum samples. This method may also be useful to identify other types of tumor-specific exosome populations with applications in the fields of cancer research and cancer diagnosis.^195^

PCa is the second most frequent cancer in men across the world.^250^ Despite its use in immunoassays, direct Raman spectroscopy is currently limited by its low signal intensity. In the 1990s, it was reported that the strength of Raman signals could be considerably boosted when the analytes were absorbed onto noble metal (Au or Ag) nanoparticles with rough surfaces. This is called SERS. Proteins, lipids, nucleic acids, and some other biological molecules show intense Raman signals in the 400–1,800 cm^−1^ and 2,800–3,100 cm^−1^ regions, allowing reasonably specific sample identification and quantification.^251^ Tian et al.^252^ used magnetic enrichment and SERS probes to develop a sandwich immunoassay to identify the trapped exosomes. The requirement for metal substrates, however, restricts applicability. Johan et al. proposed novel Raman active polymeric nanoparticles (Raman beads) that incorporated a number of alkyne, nitrile, and azido groups for multicolor Raman bioimaging in cells to effectively eliminate interfering signals originating from cell-based biomolecules. The biologically silent region of the Raman spectrum (1,800–2,800 cm^−1^), where the 18 types of Raman beads showed strong and characteristic Raman vibrations (up to 104), considerably increased the application of Raman beads. When Raman beads and microfluidics were combined, it opened up new possibilities for system miniaturization and combined the benefits of both methods. Raman analysis can now be performed automatically and reproducibly in nanoliter volumes using Raman microfluidics.^253^ Wang et al.^254^ created an *in situ* Raman assay chip that simultaneously allows consistent flow mixing and immunomagnetic separations on the same chip. Exosomes can be enriched using a staggered triangular pillar array mixing channel equipped with anti-CD63 magnetic beads. The anti-EpCAM-functionalized Raman-active polymeric nanostructures with a quantifiable signal at 2,230 cm^−1^, allow rapid (less than 1 h) detection of exosome samples. The detection limit of this biochip was around 1,600 particles mL^−1^ with a 20-μL sample volume. This microfluidics Raman chip could be a promising screening test for diagnosis of PCa. The study suggested that the microfluidics Raman chip can successfully distinguish individuals with PCa and healthy control individuals, making this strategy applicable to analysis of clinical specimens.^254^ Vaidyanathan et al.^255^ used a tunable alternating current electrohydrodynamic (ac-EHD) technology known as “nanoshearing” to construct a multiplexed microfluidics device for selective capture and identification of several types of exosomes. Electrical forces created by the ac-EHD act in the layer within nanometers of the electrode surface to produce miniaturized fluid flow. The capture specificity is improved and nonspecific adsorption of weakly bound molecules is prevented by this miniaturized liquid flow. This method allows analysis of exosomes obtained from HER2- and PSA-expressing cells as well as the isolation of exosomes from breast cancer samples. The ac-EH device showed a limit of detection (LOD) of 2,760 exosomes/L and was three times more sensitive compared with hydrodynamic flow-based analysis (LOD of 8,300 exosomes/L). The study suggests that this method might be useful for analyzing exosomes in biological samples as a simple and rapid quantitative technique.^255^ More details about microfluidics-based exosome isolation and detection methods related to human cancer cells are given in Table 2.

**Table 2:** MFs and exosomes in cancer

Strategy	Isolation method	Cancer	Sample/sample volume	Detection method	LOD	Marker detection	Reference
On-chip immuno-isolation and *in situ* protein analysis	antibody-labeled magnetic beads	NSCLC	plasma/30 μL	fluorescence	0.281 pg/mL for IGF-1R and 0.383 pg/mL for p-IGF-1R	tumor-associated markers (EpCAM, α-IGF-1R, and CA125), common exosomal markers (CD9, CD81, and CD63)	He et al.^236^
Integrated MF approach (ExoChip)	IA capture	pancreatic	serum/400 μL	fluorescence	0.5 ppm fluorescence sensitivity	Rab5 and CD63 capture exosomes	Kanwar et al.^194^
Nano-HB chip	nanostructured IA capture	ovarian cancer	plasma/2 μL	fluorescence	10 exosomes/μL	circulating exosomal CD24, EpCAM, and FRα markers to detect ovarian cancer	Zhang et al.^241^
Integrated MF approach (ExoPCD-chip)	immuno-magnetic beads	liver	serum/30 μL	electrochemical	4.39 × 10^3^ particles/mL	CD63 capture exosomes	Xu et al.^256^
ACE (angiotensin converting enzyme) microarray	AC electrokinetic capture	pancreatic	whole blood/25 μL	fluorescence	limited only by exosome concentration	Glypican-1 and CD63 capture exosomes	Lewis et al.^257^; Ibsen et al.^258^
Exodisc	filtration	bladder	urine/1 mL	colorimetric	–	CD9 and CD81	Woo et al.^259^
Double-filtration MF biochip	filtration	bladder	urine/8 mL	colorimetric	–	CD63 capture exosomes	Liang et al.^260^
Immunocapture and quantification of circulating exosomes	immuno-magnetic beads	breast	plasma/50–200 μL	fluorescence	–	CD63 and major histocompatibility complex (MHC) class I, EpCAM-positive exosomes, HER2-positive exosomes	Fang et al.^246^
Real-time, label-free profiling of CREs	IA capture	breast	serum/1 mL	surface plasmon resonance (SPR)	∼2,070 exosomes/μL	CD9/CD63-positive exosomes, HER2- positive exosomes	Sina et al.^195^
ExoSearch	immuno-magnetic beads	ovarian cancer	plasma/10 μL–10 mL	fluorescence	7.5 × 10^5^ particles/mL	three exosomal tumor markers (CA-125, EpCAM, CD24)	Zhao et al.^240^
Nano-interfaced MF exosome (nano-IMEX)	IA capture	ovarian cancer	plasma/2 μL	fluorescence	50 exosomes/μL (80 aM)	tumor-associated markers, common exosomal markers (CD63 and CD81)	Zhang et al.^261^
MF-based mobile exosome detector (μMED)	IA beads	concussion	serum, mouse/1–500 μL	fluorescence	10,000 exosomes/μL	CD45, CD61, CD81, and GluR2-positive exosomes	Suck et al.^262^
iMEX (integrated magnetic-electrochemical exosome)	immuno-magnetic beads	ovarian cancer	plasma/10 μL/marker	electrochemical	3×10^4^ exosomes	CD63, EpCAM, CD24, and CA125	Jeong et al.^263^
Nano-plasmonic exosome (nPLEX) assay	IA capture	ovarian cancer	ascites	SPR	∼3,000 exosomes (670 aM)	EpCAM, CD24, CA19-9, Claudin 3, CA-125, MUC18, EGFR, HER2, CD41, CD45, D2-40, HSP90, HSP70, CD63, and immunoglobulin G (IgG)	Im et al.^239^
Alternating current electrohydrodynamic (ac-EHD)-induced nanoshearing biochip	electrohydrodynamic immunoaffinity	prostate and breast	serum/500 μL	colorimetric	2,760 exosomes/μL	HER2, PSA	Vaidyanathan et al.^255^
AuNC-exosome-AuR	IA capture	lung cancer	urine/500 μL	dark-field microscopy (DFM)	down to 1 particle/μL	CD63, CD81, LRG1	Yang et al.^264^
Magnetic (Fe_3_O_4_NPs)-based MF	immuno-magnetic beads	pancreatic cancer	whole blood/500 μL	colorimetric	∼2 × 10^10^ exosomes	CD9 and CA19-9	Sancho-Albero et al.^265^
MF Raman chip	immuno-magnetic beads (Raman beads)	PCa	serum/20 μL samples	Raman	1.6×10^2^ particles/mL	CD63-Mag, EpCAM-functionalized Raman beads	Wang et al.^254^
ExoChip	–	NSCLC	blood/30–100 μL	–	1.47×10^9^ particles/mL	CD63, CD9, CD81	Kang et al.^266^
Ciliated MF device system	antibody-labeled ciliated micropillar	MDA-MB-231 human breast cancer	cell lines	fluorescence	–	CD63	Qi et al.^267^
On-chip microbead immunomagnetic capture	immuno-magnetic beads	breast cancer	blood/2 μL	fluorescence (colorimetric)	–	CD63, CD9, EpCAM	Chen et al.^268^
OncoBean MF	IA capture	melanoma	plasma/3 mL	fluorescence	–	CD9, MCAM (melanoma Cell adhesion molecule), and MCSP (melanoma-associated chondroitin sulphate proteoglycan)	Kang et al.^269^
Herringbone-grooved MF device (microchannels)	IA capture	ovarian cancer	serum	fluorescence	–	CD9 and EpCAM	Hisey et al.^270^
MF chip-based electrophoresis	centrifugation	cell line	serum, medium	fluorescence	–	–	Marczak et al.^271^
Mechanical forces (tunable MF system)	size-dependent purification	cancer cell line (SW620)	medium	fluorescence	–	–	Shin et al.^272^
Viscoelasticity-based MF system	continuous, size-dependent, and label-free manner	cell line/200 μL	medium, serum	fluorescence	–	–	Liu et al.^273^
Dielectrophoresis chip-based MF system	dielectrophoresis (DEP)	lung cancer	plasma/200 μL	HPLC-MS,(high-performance liquid chromatography/mass spectrometry) qRT-PCR	–	protein (CD81, EGFR), miRNA (miR-21, -191, -192), mRNA (CD81, GAPDH [Glyceraldehyde-3-Phosphate Dehydrogenase], EGFR)	Chen et al.^274^
MF filtration system (nanoporous membrane)	filtration	melanoma-grown mice	whole blood	qRT-PCR	–	CD9, CD63, CD81	Davieset al.^208^
Droplet digital ExoELISA	immunocapture by magnetic NPs	breast cancer	plasma	droplet digital ELISA	∼10 exosomes/μL (10^−17^ M)	CD63, Glypican-1	Liu et al.^275^
Exosome track-etched magnetic nanopore (ExoTENPO) chip	immunocapture by magnetic NPs	pancreatic cancer	serum	PCR, machine learning	–	CD9, CD81, EpCAM, EV-associated mRNA	Ko et al.^276^
Exosome-specific, dual-patterned immunofiltration (ExoDIF)	immunocapture by solid surface	human breast cancer cell line, MCF-7	plasma, medium	fluorescence	–	CD63, EpCAM	Kang et al.^277^
Bio-inspired NanoVilli chips	immunocapture by solid surface	NSCLC	plasma	RT-ddPCR, fluorescence	–	ROS1, T790M, EpCAM, EGFR	Dong et al.^278^
^EV^HB-Chip	immunocapture by solid surface	glioblastoma multiforme	serum, plasma	fluorescence	100 EVs/μL	Podoplanin, EGFRvIII, EGFR, PDGFR (platelet-derived growth factors)	Reátegui et al.^279^
MF immunoaffinity-based isolation of microvesicles	immunocapture by solid surface	glioblastoma	serum, medium/400 μL	scanning electron microscopy (SEM), RT-PCR	–	CD63, EV-associated total RNA	Chen et al.^280^

## miRNA biogenesis

miRNAs are short non-coding RNAs that were first discovered in 1993.^281^ Plants, mammals, and viruses have miRNAs, and they play a role in RNA silencing and post-transcriptional regulation of genes. They have also been found to be involved in cancer, neurological disorders, and other disease processes.^249^ miRNA transcriptional units can be located on the introns and exons of their specific genes or on other genes.^282^ RNA polymerase II is mainly responsible for transcription of primary miRNAs, whereas RNA polymerase III produces the remaining miRNAs. RNase III Drosha and the RNA-binding protein DGCR8 convert primary microRNA (pri-miRNA) into a precursor miRNA (pre-miRNA) of around 70 nt.^283^ The pre-miRNA then exits the nucleus using the exportin-GTPase RAs-related nuclear protein (RAN) system and is processed by Dicer to produce 22-nt double-stranded mature miRNA.^284^ This is subsequently bound by an argonaute (AGO) protein complex to form miRNA-induced slicing complex (miRISC). The RISC complex only holds one strand of miRNA, whereas the other strand, the passenger strand, is destroyed. The RISC complex regulates miRNA stability and turnover rate, which is crucial for post-transcriptional regulation of gene expression. Because of its sequence complementarity, the loaded miRNA is able to bind to its target mRNAs. If a strong match is made, then the mRNA will be degraded; otherwise it remains untranslated. miRNAs, which normally bind to the 3′UTR of target mRNAs, can target numerous different mRNAs because of their short length; any particular mRNA can bind to various miRNAs of other types.^285^

## miRNAs and cancer: from therapy to diagnosis

Calin et al.^286^ located the genetic locus of miR15 and miR16 on chromosome 13q14, a region lost in most individuals with B cell chronic lymphocyte leukemia (B-CLL), and subsequently discovered a relationship between miRNAs and cancer for the first time. Since then, instability or amplification of chromosomes in several human malignancies has been widely associated with miRNAs by other groups. Moreover, the staging, progression, and metastasis of various cancers has been associated with abnormal miRNA expression, according to many experimental and clinical investigations (Figure 7).^287^^,^^288^ miRNAs have been reported to act as tumor promoters (oncomirs) or tumor suppressors (anti-oncomirs), depending on the context.^289^ Cancer stem cells (CSCs) and epithelial-mesenchymal transition (EMT), both of which encourage cancer spread and treatment resistance, have been found to be influenced by miRNA expression.^290^^,^^291^

By suppressing tumor suppressor genes and/or genes that govern cell differentiation and death, oncomirs can encourage tumor growth. Because of gene amplification, epigenetic alterations, or transcriptional deregulation, oncomirs have been found to be upregulated in many cancers.^292^ The miR-17-92 cluster, which is found on chromosome 13q31 and is overexpressed in lung cancer and other cancers, is a well-known miRNA oncomir.^293^^,^^294^ miR-21 has been demonstrated to cause apoptosis in human glioblastoma cells by activating caspases, whereas silencing of miR-21 decreased *in vitro* and *in vivo* growth of breast cancer cells by triggering Bcl-2 down-regulation and increasing apoptosis.^295^^,^^296^

Let-7, miR-17-5p, miR-29, miR-34, miR-127, and other miRNAs have been identified as tumor suppressors.^292^^,^^297^ Interestingly, miR-15 and miR-16 were the first tumor-associated miRNAs to be established with tumor-suppressive activity.^286^^,^^298^ miR-15/16 causes apoptosis by down-regulating the anti-apoptotic gene BCL2.^299^ Another study found that miR-16 can inhibit PCa cell growth by regulating CDK1 and CDK2, two cell cycle regulatory proteins,^300^ miR-15a and miR-16-1 have also been shown to affect the survival, growth, and metastasis of PCa cells by triggering CCND1 (encoding cyclin D1) and WNT3A.^301^ The let-7 family members are well-investigated tumor suppressor miRNAs that are underexpressed in several cancers and could play a role in diagnosis and prognosis of cancer.^292^^,^^302^

On the other hand, some miRNAs have been proposed to be promising therapeutic targets because of their tumor-promoting activity. For instance, the growth of breast and stomach tumors was inhibited in xenograft mouse models when the angiogenesis regulator miR-29b was inhibited.^303^^,^^304^. Another clinically relevant miRNA, miR-34a, is down-regulated in breast cancer as well as some other cancer types. The transcription factor Fos-related antigen (Fra-1), which modulates cell death, growth, and differentiation, and the nicotinamide adenine dinucleotide (NAD)+-dependent histone deacetylase sirtuin-1 (SIRT1), which disables the tumor suppressor p53, have also been found to be targets of miR-34a.^305^^,^^306^ miR-34a was administered to a group of individuals with refractory advanced solid tumors in a phase I clinical trial along with dexamethasone and produced some clinical responses.^307^

Dysregulation of miR-15a and miR-16-1 have been associated with enhanced survival, proliferation, and metastasis, in prostate and pancreatic cancer. These miRNAs target CCND1 (cyclin D1), WNT3A, and BCL2. WNT3A is a component of the Wnt/β-catenin signaling pathway, which is involved in cell-cell adhesion as well as stimulation of oncogenic factors such as c-Myc and CCND1. miR-15a restoration led to tumor growth inhibition in an *in vivo* model of PCa, whereas miRNA-15a knockdown led to increased infiltration and proliferation in a mouse model of pancreatic cancer.^301^^,^^308^

A number of miRNAs (let-7c, miRNA-182, miRNA-205, miRNA-138, miRNA-224, miRNA-513a-3p, miRNA-34a, miRNA-106a, miRNA-31, miRNA-92b, miRNA-15b, and miRNA-27a) have been shown to target various genes involved in regulation of sensitivity to cisplatin (CDDP) (a first-line chemotherapy for lung cancer) in NSCLC.^309^ Alterations in expression of miRNA-96, miRNA-199a-5p, miRNA-182, miRNA-340, and miRNA-130a have also been found to affect the susceptibility of HCC to CDDP.^309^ ABCC2 is an ATP-dependent transport protein that boosts drug efflux and lowers intracellular CDDP concentration. ABCC2 up-regulation in tumor cells can make them resistant to CDDP, along with many other cytotoxic drugs. BCL2-like 1 (Bcl-xl), a member of the anti-apoptotic protein family, inhibits chemotherapy-induced apoptosis in cancer cells. Let-7c can target ABCC2 and Bcl-xl, leading to their down-regulation and increasing A549 cell sensitivity to CDDP.^310^ miRNA-31, on the other hand, can suppress ABCB9, another ATP-binding cassette (ABC) transport protein family member, enhancing CDDP resistance in NSCLC cells.^311^

Early stages of several cancer types have been associated with dysregulation of a plethora of circulating miRNAs that could be detected prior to any evident clinical symptoms and before any biopsy or imaging procedure. In individuals with NSCLC at stages I and II, plasma miR-21-5p, miR-223-3p, miR-141-3p, miR-145-5p, miR-155-5p, and miR-20a-5p levels have been found to be significantly elevated.^312^ Circulating miR-126-3p, miR-210-3p, miR-183-5p, and miR-182-5p have also been found to allow early diagnosis of individuals with NSCLC with sensitivity and specificity comparable with that of the classic tumor marker CEA.^313^ NSCLC stages I–IIIA could be effectively distinguished by measuring pri-miR-944 and pri-miR-3662, two primary miRNAs.^314^ In colorectal cancer serum exosomes, there were considerably lower levels of miR-125a-3p along with higher plasma levels of miR-23a-3p, miR-27a-3p, miR-376c-3p, and miR-142-5p.^315^^,^^316^ Early in the course of glioma development, plasma miR-1825-3p has been shown to be underexpressed, and its level is linked to tumor growth and poor prognosis.^317^ In stage I breast cancer, miR-4281-3p, miR-1202-5p, miR-1207-5p, miR-1225-5p, miR-4270-5p, and miR-642b-3p have been shown to be increased in the circulation.^316^ Therefore, the early stages of several cancers could be screened by detecting specific circulating miRNAs.

Biosynthesis of miRNAs, which is carried out by several components, is a complex process divided into canonical and non-canonical pathways. Cancer growth pathways, such as cell cycle disruption and immune evasion, have been found to be associated with deregulation of certain miRNAs. miRNAs are therefore emerging as important biomarkers in cancer diagnosis and prognosis because of their potential to influence dozens of cancer-related genes. DNA damage repair-associated genes, drug target-associated genes, pharmacokinetics-associated genes, and many cell signaling pathways play a role in cancer treatment resistance associated with miRNA dysregulation. There are emerging miRNA-based therapeutic approaches using miRNA inhibitors or knockdown as well as miRNA replacement treatment and miRNA mimics. miRNA therapy would likely be used together with chemotherapy, immunotherapy, or radiation treatment. miRNAs are clearly going to have a big effect on the development, diagnosis, and therapy of human cancer in the future. More research in this area is needed to fully explore the mechanisms and develop cancer-targeting medicines.

## Microfluidics and miRNAs in cancer

As mentioned above, miRNAs have become a hot topic among researchers since their discovery in 1993, especially in carcinogenesis and cancer development.^289^^,^^318^ Some miRNAs can be consistently detected in the peripheral circulation as well as in some other bodily fluids and are therefore promising as biomarkers for early-stage cancer diagnosis.^319^, ^320^, ^321^ Many of the methods used for miRNA detection and quantification, such as quantitative reverse-transcriptase PCR (qRT-PCR), microarrays, and NGS, despite their many benefits, are costly and time consuming, which makes them unsuitable for diagnosis at the POC or in rural undeveloped areas or for large-scale screening studies.^322^ Newly introduced biosensing devices based on microfluidics could solve these challenges by allowing rapid and cheap detection of miRNAs.^323^^,^^324^

A variety of microfluidics-based techniques have been used for detecting miRNAs. These include SERS,^325^ SPR,^326^ upconversion nanoparticles (NPs),^327^ rolling cycle amplification (RCA),^328^ and enzyme-assisted target recovery (EATR).^329^ Other methods include NP-based colorimetry,^330^ fluorescence resonance energy transfer (FRET),^331^^,^^332^ and electrochemical detection.^333^ Of these approaches, SERS and SPR can detect the lowest miRNA concentrations (less than 1 aM for SERS), taking advantage of surface enhancement. However, quantification of real samples is still difficult because of the lack of standardization, unknown quantities, and the poor reproducibility of SERS. Upconversion NP-based sensors rely on absorption of low-energy photons and emission of high-energy fluorescence after target capture with a detection limit of 0.1 nM, mostly governed by conversion efficiency. Colorimetric approaches leverage the unique optical features of gold nanoparticles (AuNPs), and the visible color shift of aggregated AuNPs provides a detection limit of about 1 fM. Graphene oxide and its analogs,^331^^,^^332^ MoS_2_, WS_2_, and some other newly emerging two-dimensional (2D) materials can capture single-stranded DNA and quench fluorescent signals when the molecules are in close proximity.^334^^,^^335^ When the target miRNAs have been tagged with fluorescent reporters and desorbed from the 2D materials, fluorescence emission is restored. Nanomaterial-based FRET and colorimetry are sensitive and reliable techniques, but each type of miRNA requires its own detection element, and miRNA detection could require a sample volume of a few microliters or greater, necessitating a large set of test samples to perform repeated detection. Amplification-based detection technologies, including RCA and EATR, are also available. High-throughput identification of miRNAs in a small amount of sample remains a challenge for most approaches.^336^^,^^337^ Microfluidic chips are now used with a variety of procedures and have a wide range of applications.

Jiao et al.^338^ developed a droplet-PCR-based 3D-printed microfluidics chip for miRNA identification. Kim et al.^339^ described a non-powered microfluidics chip that employs self-pumping via PDMS air uptake without requiring any additional power. Novara et al.^340^ created numerous biological detection platforms by combining a Raman detection substrate with a microfluidics chip. Modification of chemical substrates could enable microfluidics chips to become more powerful. For example, the remaining amino groups in an aldehyde-assembled^341^ substrate could be used to immobilize a DNA probe, and poly-L-lysine (PLL)^342^^,^^343^ can immobilize DNA or miRNA probes using electrostatic binding. Combination of designed biomaterials with a microfluidics chip is promising for high throughput and convenient miRNA detection. Gao et al.^344^ used three-segment hybridization and fluorescence imaging by combining PLL-modified, self-assembled slides with high-throughput microfluidics chips to develop miRNA quantitative assay technology. In this work, miR-4732, miR-k12-5, miR-3646, and miR-4484 (four typical miRNAs found in intraductal breast cancer) were employed to demonstrate the effectiveness of the biochips. To identify miRNAs, a three-segment hybridization technique was utilized, with the target miRNA binding to the capture probe at one end and a fluorescent dye at the other end. Detection probes with fluorescent labels were bound to the target miRNA on the chip. Then the capture probes were immobilized on the device by a miRNA hybridization probe. Finally, a laser scanner was employed to excite the fluorescence signal of the capture probe-miRNA-detection probe complex on the chip and to eliminate non-specific probe binding.

**Figure 8:**
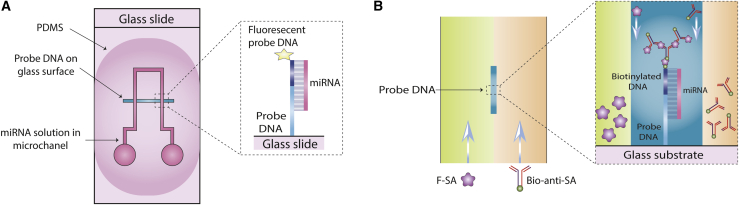
chip-based approaches (A) The PF MF device in which PDMS absorbs air in the outlet chamber, making it a self-stand pumping device. Probe DNA is immobilized on the glass surface, microchannels convey the sample to the probe, and miRNA hybridization and detection take place. (B) An enlarged view of a laminar flow in the microchannel. The laminar flow conveys fluorescein isothiocyanate (FITC)-labeled streptavidin (F-SA) and biotinylated anti-streptavidin (B-anti-SA). Sandwich hybridization and dendritic amplification take place at the intersection between the probe DNA-patterned surface and the interface of the laminar flow. This figure was adapted from other studies.^341^^,^^346^^,^^347^

The lab-on-a-chip (LOC) approach has become a hot topic among microfabrication scientists over the previous two decades, attracting major industry attention for future biomedical applications. Economical, rapid, and combinatorial detection of biological materials are the main advantages of LOC systems. These properties of LOCs have encouraged researchers to develop innovative devices that could be used for consumer applications as POC systems.^344^ Development of POC systems requires a miniaturized electronic readout, which could be based on MicroTAS (micro-total analysis system) or MEMS (micro-electro-mechanical system).^24^^,^^345^ Arata et al.^341^ described a power-free microfluidics device requiring a small sample size (Figure 8A) that could be used as a POC system for miRNA identification. The device is powered by degassed PDMS, requiring no external energy source for fluid pumping. Signal amplification in the microfluidics device relies on laminar flow-assisted dendritic amplification (LFDA).^346^ The miRNA is identified using a sandwich hybridization approach in which a miRNA capture probe is mounted on the glass surface, and the sample is transported to the immobilized probe through the microchannels. Subsequently, the miRNA is hybridized to the probe, and LFDA amplifies the signal (Figure 8B). This device is promising for further development of miRNA POC detection systems.^347^ Zhang et al.^348^ described a bacterial pathogen detection system (with a detection limit of 200 cells/L) using a manual, electricity-free centrifugal microfluidics chip with sample multiplexing and molecular label detection ability. Relying on top-spinning technology, this device could be used in rural communities as a POC tool. This microfluidics disc can carry out multiple procedures, from nucleic acid purification through target amplification and diagnosis. Centrifugal mixing of the pre-loaded reagents is used for reagent manipulation. The loop-mediated isothermal amplification (LAMP) reaction is carried out between 30°C and 60°C, a relatively low temperature produced by a mobile pocket warmer, and the fluorescence signals are detected by a tiny UV lamp. It is critical to improve the system function to the extent that it can compete with the gold-standard technology. Salim et al.^349^ employed a microfluidics miRNA-based POC device to further investigate the LAMP reaction. A fluorescence reader is incorporated into this system. It can detect and monitor miRNA-21 levels in blood samples from individuals with breast cancer in half an hour, using miRNA molecular beacon probes.^349^ Molecular beacon probes were developed to conduct screening of multiplex miRNAs at different levels within a single test, which improves the true positive and true negative rates and is not interfered with by other nucleic acids. miRNA-21 levels in the serum of individuals with breast cancer could be used as a biomarker; it has been shown to be as much as four times higher compared with its levels in healthy individuals. This POC device was used to evaluate 51 blood samples, 30 from healthy control individuals and 21 from individuals with breast cancer.^349^ qRT-PCR was used to verify the effectiveness of the device. It has been suggested that this test could be used in rapid staging of cancer, required by physicians who want to select a treatment plan.

A microfluidics paper-based analytical device (PAD) was used for miRNA analysis. Fluid flow on a PAD can be controlled by capillary forces in a direct manner not requiring any external driving forces.^350^^,^^351^ Cellulose paper allows a variety of chemical and biochemical reactions and provides passive liquid transfer.^352^ Being hydrophilic, paper may be thought of as a porous material with a homogeneous structure that permits useful reactions.^353^ PADs are highly disposable because of their minimal weight, cheap cost, and low sample/reagent consumption.^354^ MicroPADs (μPADs) have evolved into useful tools for analyzing nucleic acids and other biological molecules.^355^ Detection techniques such as colorimetric detection and fluorescence are commonly used in μPADs.^356^^,^^357^ Among these detection strategies, colorimetric detection is a rapid and easy way of detecting samples.^358^^,^^359^ The results are plainly visible with the naked eye.^360^ Fluorescent samples can be sensitively analyzed by a fluorescence detection technique, which minimizes interference with the sample. The detection method of laser-induced fluorescence (LIF) is a specific type of fluorescence detection, well suited for μPADs.^361^^,^^362^ It should be borne in mind that the number of different intracellular miRNAs might be 1,000 or even higher.^363^ Amplification or enrichment techniques play a major role in allowing miRNA detection by standard methods. DSN (duplex-specific nuclease) is an enzyme that can cleave each single DNA chain in double-stranded nucleic acids in a selective manner. DSN can cleave TaqMan probes, DNA chains, releasing fluorophores into solution and increasing the fluorescence emission intensity when these DNA sequences combine with target miRNAs.^364^ Because the content of miRNAs can be as low as 1,000 molecules in each cell,^363^ it is impossible for ordinary detection methods to directly determine cellular miRNAs without amplification or enrichment. The sensitivity of miRNA detection can be significantly improved using DSN amplification.^365^ In addition, DSN amplification does not require any complicated procedures and is relatively fast, making it a useful approach for miRNA analysis. DSN amplification on μPADs, however, is significantly more challenging than in test tubes. Paper has a large specific surface area, and the fibers may bind strongly to nucleic acids; therefore, a substantially higher number of miRNAs will be required for detection. In addition, the reagent storage shelf life and fluorescence signal obtained on paper differ from those obtained in clear solutions. Researchers have recently developed a microfluidic DSN paper-based LIF sensor to selectively detect miRNAs in cancer cells with good sensitivity.^366^ Sample detection on the paper-based device was initially performed using LIF detection. DSN (0.10 U) and TaqMan sensors (0.25 μM) were added to the folded paper circle (diameter, 4 mm) chip and could be stored under ideal conditions. Using cyclical digestion of the hybrids between miRNAs and TaqMan probes by DSN enzyme, the fluorescence signal was amplified after addition of the miRNA solution. The entire procedure, including sample heating, could be completed in about 40 min. miRNA-21 and miRNA-31 had detection limits of 0.20 and 0.50 fM, respectively, leading to miRNA consumption of just 1.0 or 1.5 zmol. The approach demonstrated high specificity when tested with mismatched miRNAs. The concentrations of miRNA-21 and miRNA-31 in lysates of A549 and HeLa cancer cells, along with LO2 hepatocytes, were successfully measured by this approach. miRNA-21 and miRNA-31 concentrations in HeLa cell lysates (3.75 × 10^4^ cells/mL) were 1.74 × 10^−13^ M and 6.29 × 10^−14^ M, respectively, and 3.07 10^−15^ M and 3.28 × 10^−15^ M in A549 cell lysate (8.33 × 10^6^ cells/mL). The recovery rates varied from 87.30% to 111.83%, verifying the study findings. miRNAs could be efficiently and sensitively determined in cancer cells in a selective manner.^366^

Other groups introduced a new miRNA detection method by designing a rapid and affordable microfluidics chip.^341^^,^^346^^,^^367^ There were two main aspects of this strategy. First, pumping was accomplished by degassed PDMS, which is widely used as a microfluidics chip material, so no external power source was needed.^368^ Second, the process was enzyme free because of fluorescence signal amplification on the microfluidics platform using an LFDA method.^369^^,^^370^ The duration of the detection process was claimed to be as short as 20 min.^346^ With a sample volume of only 0.5 μL, the LOD was 0.5 pM. This method has been reported to be able to simultaneously detect miR-16, miR-21, and miR-500.^367^^,^^371^

A surface-functionalized, power-free microchip (SF-PF microchip) for POC testing has been introduced as a rapid and convenient biomarker detection system.^372^^,^^373^ Electron beam-induced graft polymerization (EB grafting) and chemical modification of the inner surfaces of the microchannels were used to create this PF microchip. The pump-free microchip was powered by PDMS gas solubility rather than electric power.^368^^,^^374^ A DNA capture probe was attached to the grafted polymer chain on the microchannel surface of the PF microchip, enabling triple miRNA detection on the SF-PF microchip. The required sample volume, assessment time, and detection limit for miRNA were determined to be 1.0 μL, 20 min, and 5.0 nM, respectively.^373^ UV light-induced graft polymerization (UV grafting) on the PDMS PF microchip was used to produce a cancer biomarker SF-PF microchip for identification of hsa-miR-500a3p (miR-500). Rapid and sensitive detection with a small sample volume was obtained. The sample volume, detection time, and LOD were 0.5 μL, 18 min, and 41 fM, respectively. This LOD was 19-fold lower than the SF-PF microchip produced by EB grafting and fell within the miRNA concentration range in blood. Compared with EB grafting, UV grafting allowed easier production of the SF-PF microchip and more sensitive miRNA detection. Under normal atmospheric conditions, the SF-PF microchip could be stored for over 7 days.^374^

A summary of studies on microfluidic-based miRNA detection methods in human cancer is given in Table 3.

**Table 3:** MFs and miRNAs in cancer

Strategy	Isolation method	Cancer	Sample/sample volume	Detection method	LOD	miRNAs	Reference
Double-layered MF biosensing chip	capture DNA probe	breast cancer	serum/2 μL	fluorescence	0.146 aM	miR-125, miR-126, miR-191, miR-155, miR-21	Chu et al.^375^
Self-priming MF chip and DSN	capture DNA probe	human breast cancer cell line	medium/20 μL	fluorescence	45.35 pM	miR-100, miR-155, Let-7a	Zou et al.^376^
DNA-field-effect transistor (FET) biosensor-based MF system	DNA-FET biosensors	breast cancer	serum	–	84 and 75 aM	miR-195, miR-126	Huang et al.^377^
Integrated MF platform	antibody-coated magnetic beads	ovarian cancer	plasma	–	1.4 aM	miR-21	Sung et al.^378^
Integrated MF platform	field-effective transistor biosensors	breast cancer	plasma	–	1 fM	miR-195	Huang et al.^379^
Magnetic hyperthermia on chip	DNA hybridization on core-shell NPs	liver	plasma/0.24 μL	electrochemical	–	miR-122	Horny et al.^380^
Electrochemical MF multiplexed biosensor	CRISPR-biosensor	brain cancer	serum/0.6 μL	electrochemical	2–18 pM	miR-19b, miR-20a (from miR-17–92 cluster)	Bruch et al.^381^ and Bruch et al.^382^
Surface-enhanced Raman scattering (SERS)- MF approach	RSA probes	breast cancer cell line	medium/–	SERS spectroscopy	2.32 fM, 40 min	miR-141	Ma et al.^383^
SF-PF MF chip	capture DNA probe	cancer cell	medium/0.5 μL	UV light	41 fM, 18 min	miR-500a-3p	Ishihara et al.^374^
Hydrogel-based colorimetric assay	biotinylated DNA probe-loaded nanogold-streptavidin	cancer cell	total RNA samples	colorimetric	260 fM,	Let-7a, miR-145, miR-21	Lee et al.^384^
Poly-L-Lysine (PLL) substrate is integrated with MF chips	capture DNA probe	breast cancer	serum	fluorescence	1 pM, 30 min	miR-4732, miR-k12-5, miR-3646, and miR-4484	Gao et al.^344^
MF paper-based analytical device (μPAD)	amplification for miRNA on μPAD (miRNA sequences, TaqMan probe, and DSN)	cancer cells	–	LIF detection	0.2 fM (miR-21), 0.5 fM (miR-31)	miR-21, miR-31	Cai et al.^366^
MF exponential rolling circle amplification (MERCA) platform	capture probe	cancer cells	total RNA	fluorescence	<10 zmol levels	miR-21, let-7	Cao et al.^385^
PF MF chip	capture DNA probe (LFDA)	cancer cell	0.5 μL	fluorescence	0.045 pM (miR-196a) and 0.45 pM (miR-331), 20 min	miR-196a, miR-331	Kim et al.^339^
MF platform	capture DNA probe	lung cancer cell	–	fluorescence	–	miR-21, miR-486	Arata et al.; Allahverdi et al.^346^^,^^386^
MFs PADs	electrochemical probe (cerium dioxide - Au@glucose oxidase (CeO2-Au@GOx))	cancer cell	serum/–	electrochemical	0.434 fM	miR-21	Sun et al.^387^
Integrated droplet MF system	DNA hybridization chain reaction (real-time droplet assay)	breast cancer cells (MCF-7, MDA-MB-231)	medium	fluorescence	–	miR-21	Guo et al.^388^
MF VAL-DESI (voltage-assisted liquid desorption electrospray ionization-tandem)-MS/MS	capture DNA probe	–	miRNA-containing samples (25 μL)	mass spectrometry	0.25 pM	miR-21	Li et al.^389^
Theranostic one-step RNA detector; MF disc	capture-target-NP labeled probe	–	plasma and human cerebrospinal fluid	electrochemical	1 pM	miR-134	McArdle et al.^390^
MF TaqMan array cards	qRT-PCR-based technology	pancreaticobiliary tumors	tissue	–	–	miR-135b, −148a, −155, −196a, −210, −217, −203, −375, −1246	Gress et al.^391^
MF platform	miRNA probe	breast cancer	serum	fluorescence	–	miR-21	Salim et al.^349^
MF platform	molecular beacon (MB) probe	–	–	fluorescence and SERS reporter	10^−8^ M	miR-21	Wang et al.^392^
Droplet MF combined with ICSDP (isothermal circular-strand-displacement polymerization)	MB probe	–	0.2–1 μL	fluorescence	–	miR-210	Giuffrida et al.^393^
Digital MF devices	MB-assisted ICSDP	–	20 nL droplets	fluorescence	–	miR-210	Giuffrida et al.^394^

## Conclusion

Since microfluidics was first introduced to biological research two decades ago, the field has continued to be widely developed. Microfluidics has great potential in the diagnosis and molecular understanding of cancer, a leading global cause of death, because of its affordability, sensitivity, spatiotemporal control, and low sample consumption. The microscale microfluidics platform has made many contributions to basic sciences (e.g., physics, biology, chemistry, and physiology). The high portability of microfluidics devices has made them promising for POC diagnostics. Cancer management and biological understanding could be significantly improved through application and development of microfluidics approaches. Despite the popular assumption that microfluidics are a “cost-effective” alternative to conventional benchtop instruments, the actual costs of microfluidics devices for isolation and analysis of exosomes and miRNAs can vary widely, depending on the methodology employed. However, the continuing improvements being made in microfluidics hold great promise for design of new commercial gadgets for liquid biopsy analysis. It is predicted that samples will be analyzed for their miRNA and exosome composition in POC and clinical settings. To achieve this, a number of challenges have to be solved. First, there are insufficient clinical studies, particularly large cohort trials. Second, there is a lack of unified standardization, meaning that robust methodical reproducibility and the real potential of miRNA and exosome-based microfluidics have not been established. Third, the devices will need to achieve regulatory approval for clinical applications. Despite the recent initial approval of commercial microfluidics-based instruments for miRNA and exosome detection, we suggest that microfluidics technology will be mainly commercialized in the academic research market.

## Availability of data and material

The primary data for this study are available from the authors upon request.
